# Copper, Iron, Cadmium, and Arsenic, All Generated in the Universe: Elucidating Their Environmental Impact Risk on Human Health Including Clinical Liver Injury

**DOI:** 10.3390/ijms25126662

**Published:** 2024-06-17

**Authors:** Rolf Teschke

**Affiliations:** 1 Department of Internal Medicine II, Division of Gastroenterology and Hepatology, Klinikum Hanau, 63450 Hanau, Germany; rolf.teschke@gmx.de; Tel.: +49-6181/21859; Fax: +49-6181/2964211; 2 Academic Teaching Hospital of the Medical Faculty, Goethe University Frankfurt/Main, 60590 Hanau, Germany

**Keywords:** arsenic, cadmium, copper, drug-induced liver injury, iron, hemochromatosis, herb-induced liver injury, oxidative stress, reactive oxygen species, RUCAM, toxic liver injury, Wilson disease

## Abstract

Humans are continuously exposed to various heavy metals including copper, iron, cadmium, and arsenic, which were specifically selected for the current analysis because they are among the most frequently encountered environmental mankind and industrial pollutants potentially causing human health hazards and liver injury. So far, these issues were poorly assessed and remained a matter of debate, also due to inconsistent results. The aim of the actual report is to thoroughly analyze the positive as well as negative effects of these four heavy metals on human health. Copper and iron are correctly viewed as pollutant elements essential for maintaining human health because they are part of important enzymes and metabolic pathways. Healthy individuals are prepared through various genetically based mechanisms to maintain cellular copper and iron homeostasis, thereby circumventing or reducing hazardous liver and organ injury due to excessive amounts of these metals continuously entering the human body. In a few humans with gene aberration, however, liver and organ injury may develop because excessively accumulated copper can lead to Wilson disease and substantial iron deposition to hemochromatosis. At the molecular level, toxicities of some heavy metals are traced back to the Haber Weiss and Fenton reactions involving reactive oxygen species formed in the course of oxidative stress. On the other hand, cellular homeostasis for cadmium and arsenic cannot be provided, causing their life-long excessive deposition in the liver and other organs. Consequently, cadmium and arsenic represent health hazards leading to higher disability-adjusted life years and increased mortality rates due to cancer and non-cancer diseases. For unknown reasons, however, liver injury in humans exposed to cadmium and arsenic is rarely observed. In sum, copper and iron are good for the human health of most individuals except for those with Wilson disease or hemochromatosis at risk of liver injury through radical formation, while cadmium and arsenic lack any beneficial effects but rather are potentially hazardous to human health with a focus on increased disability potential and risk for cancer. Primary efforts should focus on reducing the industrial emission of hazardous heavy metals.

## 1. Introduction

Heavy metals like copper, iron, cadmium, and arsenic have been formed at various molecular levels from helium and hydrogen in the universe via nuclear fusion in stars during supernova explosions long before they arrived at our globe between ten and twenty billion years ago, likely and more specifically around 13.7 billion years [1,2,3,4,5]. In line with other heavy metals, the chemicals copper, iron, cadmium, and arsenic are defined as metallic elements according to their density of >5 g/cm^3^ or by considering their atomic weight of >20, but there still is ongoing uncertainty and discussion as to how best to define heavy metals and how toxicity may be incorporated in the definition [6]. In chemistry, arsenic may also be seen as a metalloid, an unprecise term for an element with characteristics between a typical metal and a typical non-metal [6,7]. Metals cannot be broken down and are non-biodegradable unless the organism detoxifies the metal ions by binding the active element with a protein or by depositing them in intracellular granules for long-term storage [6]. 

Arriving at the earth long predating human existence, heavy metals subsequently became part of the environment preceding and after human evolution. Additional exposure to heavy metals found increasingly in the ecosystem due to mankind and industry activities caused health concerns and led to considerations on how best to meet these ecology issues [6,7]. In addition to providing occupational safety through protecting workers of industry facilities releasing heavy metals, special attention was paid to re-naturize polluted soils by growing plants that take up these heavy metals [6]. However, this approach helped the soil to remove the heavy metals and reduced the local health risk, but at the same time it created the next problem of properly disposing of the contaminated plants. As a result, heavy metals continue to circulate in our ecosystem without the chance of final disposal.

As expected, some heavy metals may impair human health [6,7] and injure the liver as the central metabolic organ, which takes care of many swallowed potentially toxic substances and chemicals like heavy metals. They leave the intestinal tract via the portal system and flow through this organ [8,9]. The human liver has a critical role in maintaining somatic and cellular equilibrium and ensuring hepatic homeostasis necessary for healthy life. The focus of hepatic homeostasis is generally on metabolism [8,9,10,11,12], clearance [11], and immunity [12]. Through these processes, the liver cares for the intermediate metabolism of lipids, carbohydrates, and proteins [13], as well as for drug metabolism [14,15,16] and for the degradation of other exogenous chemicals [17,18,19,20,21,22,23]. Some of these metabolic pathways may be disturbed if the homeostasis of heavy metals is impaired.

Heavy metals are known for their specific environmental issues [24,25,26,27] and clinical challenges related to diseases of the liver [2,28]. More specifically, and in line with the U.S. Food and Drug Administration (US FDA), concern has been expressed that dietary exposure to heavy metals was recognized as a public health issue, focusing among others on arsenic and cadmium [29]. As a consequence, these two chemicals were among the four heavy metals that were intensively discussed in the current analysis. Collectively, various exogenous compounds like heavy metals are classified as risky injurious chemicals if entering the human body, where they may cause health hazards and liver injury that require special attention [2,26,27,28]. In trace amounts, a few heavy metals like chromium, copper, iron, manganese, molybdenum, selenium, and zinc are essential elements [7], initially required for human evolution on earth and subsequently needed to sustain human health. Even at trace levels, other heavy metals are diametrical to human health including to the liver of individuals, who for instance, cannot provide physiological homeostasis due to genetic reasons as evidenced by high amounts of these metals detected in their body.

This article provides a critical analysis and overview on the potential environmental and clinical risks posed by the three heavy metals copper, iron, and cadmium, as well as the metalloid arsenic. These four elements were chosen for this article because they are commonly found as contaminants in the environment and can easily enter the human body via the food chain. Clinical challenges in diagnosis and tentative mechanistic and molecular steps of the cascade of events will be discussed in detail.

## 2. Basic Considerations

In toxicology studies, the terms of toxicant and toxin are often used interchangeably despite differences in their origin [30,31]. More specifically, toxicants are any injurious substances, which are man-made, occurring in the nature, and found as contaminants in the air, soil, water, or food, whereby heavy metals commonly belong to this category of toxicants [30]. As opposed to this, and without reference to heavy metals, a toxin in a narrower sense is a natural poison classified as a secondary metabolite produced by living organisms themselves, but not just acquired from outside sources, and that typically is not harmful to the organisms themselves but can have an impact on human or animal health when consumed [1,2]. Common sources of such toxins include poisonous plants, fungi, algae, bacteria, and marine biotoxins, while the diversity of these biological systems presents challenges to analytical chemists and food safety implications [30]. In a broader sense, both forms of poisonous chemicals are often termed uniformly as toxins, used also in this article. Evaluating the possible role of heavy metals on human health risks including liver injury requires the consideration of several challenges. Some general aspects regarding heavy metals are additionally worth mentioning.

First, copper, iron, cadmium, and arsenic are commonly found in the environment together with other heavy metals or industrial toxins [7,24,25]. The combined occurrence makes causality assessment for one or the other culprits difficult and requires careful clinical analysis and extension to environments polluted by a single chemical like in the vicinity of a specific industry complex manufacturing products with a predominance of a single chemical.

Second, definitions of health and its associated parameter of disability-adjusted life years (DALYs) are often vague and differ among countries and regions. Respective results may not be applicable throughout the world.

Third, exact quantitative data on exposure to an assumed chemical culprit may hardly be obtainable. This is why in most publications respective results were not reported. 

Fourth, a major hurdle is the robust determination of the non-toxic levels of the heavy metal under consideration including arsenic and cadmium [29]. In this report, terms such as daily maximum safe exposure, oral reference dose, provisional total daily intake, or minimal risk level were differently used among various agencies from different countries, and reported reference values substantially differ from one regulatory agency to another and from one country to another [29]. Published values were often adapted by reduction within a few years due to newly incoming data. Overall, there is not enough research to know at what heavy metal levels one should expect non-cancer or cancer health problems of any exposed organ or tissue system.

Fifth, human liver injury due to heavy metals exposure was often insufficiently defined. These challenges apply to all four metals at variable extents and different levels. As a result, to circumvent redundancy in the following chapters, key points ahead are discussed below.

### 2.1. Laboratory Criteria of Liver Injury

In clinical and ambulatory settings, abnormal liver tests (LTs), like slightly increased serum alanine aminotransferase ALT), aspartate aminotransferase (AST), and alkaline phosphatase (ALP) activities, are commonly found and mostly lacking any clinical relevance, viewed as liver adaptation or tolerance [32,33]. In analogy to drug-induced liver injury (DILI) and herb-induced liver injury (HILI), mainstream international consensus among experts exists that the diagnosis of the liver injury requires threshold values of at least two liver values with ALT or AST ≥ 5 times the upper limit of normal (ULN) and/or ALP ≥ 2 times the ULN, both enzymes are viewed as typical diagnostic parameters of liver injury [32,33,34,35,36,37]. On the other hand, total bilirubin as a parameter of liver function when conjugated bilirubin is predominant, is explicitly not part of the diagnostic liver injury algorithm [33] because unconjugated hyperbilirubinemia is not specific for liver injury because it can be seen in patients with the genetic Gilbert syndrome and has a frequency of up to 8% in the general population [38]. Cases with serum ALT or ALP activities below the thresholds are not considered as liver injuries that could carry a risk for patients but are categorized as liver adaptation or liver tolerance [39]. 

### 2.2. Types of Liver Injury

The determination of the individual liver injury pattern, also known as phenotype, caused by copper, iron, cadmium, and arsenic is actually derived from criteria previously established for hepatocellular injury and cholestatic or mixed liver injury due to potentially hepatotoxic drugs and herbs [32,33]. This can easily be achieved through the calculation of the ratio (R) by using the multiples of the upper limit of normal (ULN) for serum ALT divided by ALP. Thus, R ≥ 5 classifies the hepatocellular injury, R ≤ 2 the cholestatic injury, and 2 < R < 5 the mixed liver injury. As the determination of the liver injury pattern by the R value requires only the routinely available results of serum ALT and ALP activities, this approach saves financial resources, does not require an invasive and risky liver biopsy, and is helpful for the description of clinical DILI features.

In general, experimental liver injury by intoxicating exogenous heavy metals is dose dependent, and predictable. Respective mechanistic studies in diseased individuals are scarce because the liver is a secret-keeping organ not really accessible for direct analysis. In addition, parameters in the blood that could reflect pathogenetic processes in the liver are largely missing, especially since there is a lack of circulating humoral immune markers like proinflammatory cytokines, including IL-2, IL-4, IL-6, IL-10, TNF-α, and IFN-ɣ in cases of clinical liver injury due to heavy metals [25], which are valuable data like in cases of idiosyncratic DILI [40]. 

Depending on the type of the heavy metal, the liver injury may be variable and more specific. If applicable, this is outlined in detail for individual heavy metals as described under the respective chapter. Injury specifics mostly prevail in patients with heavy metals stored in their liver like in Wilson disease or hemochromatosis. 

### 2.3. Diagnostic Approaches and Causality Assessment

**Figure 1:**
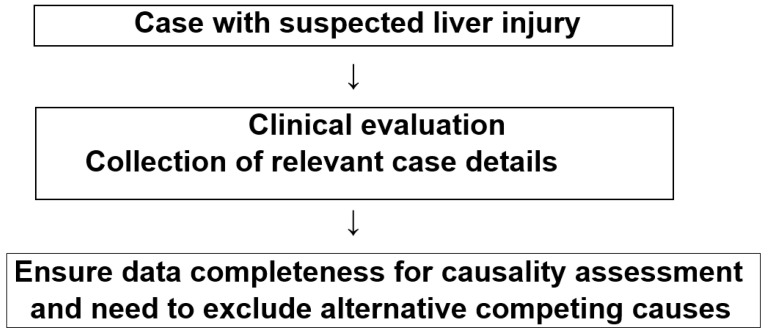
Flow chart with checklist of differential diagnoses in cases of suspected liver injury due to copper, iron, cadmium, and arsenic.

In each patient with suspected liver injury due to copper, iron, cadmium, and arsenic, a careful clinical evaluation including past medical history, data on exposure to the heavy metal, and the prospective collection of case details including complete laboratory results is essential (Figure 1).

### 2.4. Exclusion of Alternative Causes

In any patient with suspected liver injury by toxins like copper, iron, cadmium, and arsenic, although with regrets rarely done, a firm diagnosis of the heavy metal implicated in the liver injury is needed for an adequate evaluation of the case. Increased LTs signify the presence of a liver injury but do not give any information of its cause, because they are found in many other hepatic and extrahepatic as well as systemic diseases. Thus, the exclusion of competing causes is essential (Table 1).

Under ideal clinical conditions, the updated Roussel Uclaf Causality Assessment Method (RUCAM) should be used to determine causality gradings based on a final RUCAM score: ≤0, excluded causality; 1–2, unlikely; 3–5, possible; 6–8, probable; and ≥9, highly probable [33]. RUCAM was inaugurated to help establish the difficult diagnosis of DILI by a sophisticated diagnostic algorithm considering key elements of DILI that had to be scored [32], an approach in line with principles of artificial intelligence (AI) [41]. So far, RUCAM was applied for drugs causing human liver injury in 81,856 cases of DILI published 1993 until mid-2020 [42] and in a single report on intoxication with mercury and drugs [25,43] but not for any other heavy metal [25]. 

### 2.5. Heavy Metals in Plants and Horizontal Transfer

**Figure 2:**
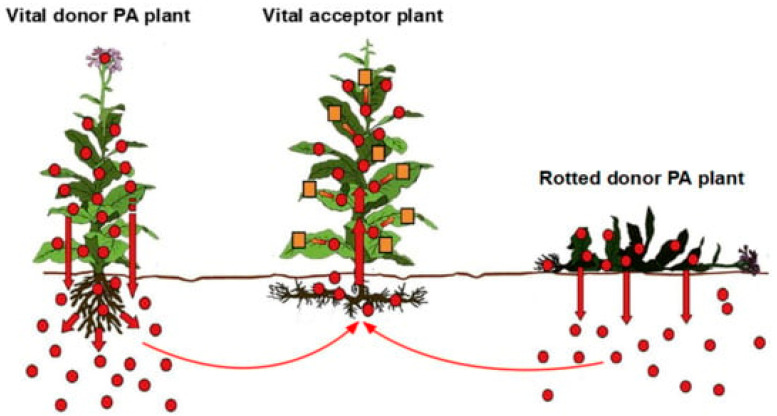
Horizontal transfer system of chemicals in plants, shown here for pyrrolizidine alkaloids as an example for heavy metals. In this analogy, any vital acceptor plant can uptake heavy metals from polluted soil and potentially serve as a vegetable for food or grain for bread. Derived from a previous open access report [45]. Abbreviation: PA, pyrrolizidine alkaloid. This figure was modified and adapted from previous illustrations and suggestions of the group of Selmar [46,47].

Heavy metals enter the human body via inhalation, skin, and the gastrointestinal tract through both the solid food chain and drinking water [6,44]. With respect to the food chain, contaminated plants are only one category among many sources. Such plants may be used as vegetables or as feeding material for grazing cattle. Plants grown on soils contaminated with heavy metals are a neglected topic but of potential clinical relevance that needs further attention in the future [23], including their use in herbal medicine [24,45,46,47,48,49]. Plants receive heavy metals from contaminated soil via their roots or rhizomes in a similar way to that described for pyrrolizidine alkaloids (PAs), through a process termed horizontal transfer system (Figure 2) [45].

Special care should be addressed to patients under a therapy with Ayurvedic medicine, commonly consisting of several herbs that may be contaminated with arsenic [50]. Contamination is viewed as the intentional adulteration of the medicinal herbs, having been grown on soil previously fortified with arsenic in the erroneous belief that it enhances therapeutic efficacy. Apart from lacking proven efficacy, problems emerge if liver injury is detected, attributable to arsenic or the herbal mixture. 

### 2.6. Issue of Experimental Studies

Mechanistic steps leading to liver injury have largely been investigated in animal models using excessive amounts of the heavy metals, but this approach neglects genetic basics, such as in patients with a hereditary hepatic accumulation of heavy metals, and is therefore less suitable for this special clinical study cohort and in line with considerations on other human liver diseases [51]. 

## 3. Copper

Copper (Cu, from the Latin: cuprum) is a typical heavy metal environmentally found on earth [2,52,53]. It is required as a trace element for cellular homeostasis in the human liver and various other organs. This metal is also essential for other living organisms such as animals and plants. In humans, copper levels in the physiologic range are beneficial, but lower or higher amounts commonly create health problems, with the exemption of higher amounts used as a rare therapy for patients with cancer. 

### 3.1. Physiology

Copper enters the body via the intestinal tract, facilitated by a transporter protein in the mucosa cells of the small intestine, known as copper membrane transporter 1 (Ctr1; SLC31A1) [2], that is also important for copper homeostasis in the liver. This intestinal transporter helps carry copper inside the intestinal cells where part of the copper is bound to metallothionein, and part is carried by the antioxidant protein 1 (ATOX1) to an intestinal organelle system known as the trans-Golgi network (TGN). In the case of rising copper levels within intestinal cells, an intestinal protein called ATP7A releases copper into the portal vein to the liver. Liver cells carry the CMT1 protein and metallothionein, and then hepatic ATOX1 binds the copper inside the hepatocytes. Once here, ATP7B links the copper to the hepatocellular TGN, from which the copper is incorporated in cytoplasmic vesicles near the cell periphery to be released into the bloodstream and the bile, thereby removing the excess copper [2]. Apart from the liver, injurious effects of copper in high concentrations may be helpful in future cancer therapy due to a process called cuproptosis, because a correlation was found between cancer angiogenesis, proliferation, growth, and metastasis and the accumulation of copper ions [54,55,56]. 

Available in the oxidized state as Cu(II) or reduced state as Cu(I), the heavy metal helps play a significant role in cellular physiology as a catalytic cofactor in cell redox systems of enzymes [53]. Copper is needed by the body, functioning as a cofactor for enzymes such as ceruloplasmin, cytochrome c oxidase, dopamine beta-hydroxylase, superoxide dismutase, and tyrosinase [2]. In addition, copper participates in the hepatic mitochondrial respiratory chain and intestinal absorption of iron as another heavy metal [53]. 

The average daily intake of copper by human adults assessed via a process of balance studies should be regarded with caution because of uncertainties regarding copper concentration in various foods and water as well as concern as a result of the homeostatic mechanisms controlling copper absorption and excretion [57], providing sufficient amounts of copper for blood [58] and liver [59]. Although the beneficial effects of copper as an essential trace element are well known, there is uncertainty with respect to copper reference values for humans due to different recommendations by various national authorities [57]. However, some proposals may be summarized for copper reference ranges as follows [58,59]: blood free serum copper: 1.6–2.4 μmol/L or 10–15 μg/dL [59]; blood total copper: 10–22 μmol/L or 63.7–140.12 μg/dL [58]; serum ceruloplasmin: 2.83–5.50 μmol/L or 18–35 μg/dL [59]; total 24 h urine copper 0.3–0.8 μmol or 20–50 μg [59]; and liver copper 0.3–0.8 μmol/g of tissue or 20–50 μg/g of tissue [59]. On a quantitative basis it has been assumed that normal urinary copper excretion/reabsorption is <70 µg/d and fecal copper output accounts for 1–2 mg/d, originating from the bile [60]. 

The determination of copper levels can assist in diagnosing several disease processes. These conditions may be monitored by looking at the total copper, the free serum copper, 24 h urine copper, and liver biopsy copper concentrations. Serum ceruloplasmin is also a valuable test and can be used to determine the free serum copper [57,58,59]. 

### 3.2. Copper as Pollutant and the Issue of Health Hazards

#### 3.2.1. Sources of Copper as an Environmental Pollutant

**Figure 3:**
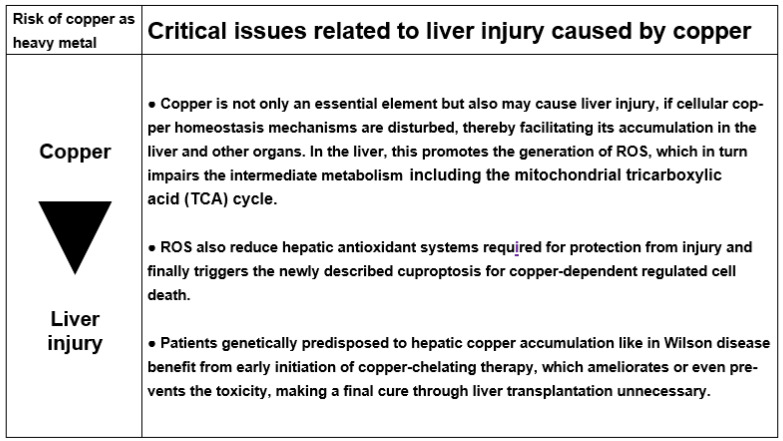
Issues connected to liver injury due to copper. Abbreviation: ROS, reactive oxygen species.

Copper is commonly found in the environment [25], such as in and around solid waste fills [23], soil [23,61,62], drinking water [63], and the atmosphere [61]. As an antifungal chemical, copper is widely used in agriculture, explaining its detection in edible plants [64] and in plants used to prepare herbal medicines [24,49]. A prerequisite for the contamination of plants with copper is that they were cultivated on contaminated grounds and had access to contaminated water [64], facilitating copper acquisition by the plants via horizontal copper transfer similar to the uptake of pyrrolizidine by plants (Figure 3). The long-distance transport of the plants may cause external contamination of the plants [64], based on the observation that urban vehicle traffic causes contamination with copper and other heavy metals in road sweeping waste and bottom sediments of retention tanks [65].

Similarly, and most importantly in the clinical context of Wilson disease, products containing high amounts of copper like dark chocolate and cocoa should not be consumed by patients with Wilson disease [66]. This is in line with early observations that chocolate contains copper in amounts up to 16.50 ± 1.29 µg/g of a chocolate with 85% cocoa, and is associated with a linear correlation between the copper content of the chocolate and its cocoa content and a correlation coefficient of 0.89, showing that the cocoa largely contributed to the copper in the chocolate [67]. Raw cocoa beans from plantations in Nigeria had copper contents ranging from 104 µg/g to 642 µg/g, attributed to the use of copper sulfate as a fungicide for disease prevention on these plantations and detected in soil and vegetation components [68]. It seems plausible that the high copper levels detected in cocoa [68] (Owena cocoa, *Theobroma cacao* L.) are due to direct contact with the fungicide or via uptake from the soil by horizontal transfer similar to plant pyrrolizidine alkaloids (Figure 2). 

Copper is used in agriculture [69,70], with preference in viticulture to protect grapes from downy mildew [70] and with copper detected in agricultural land focusing on soil surrounding ponds [62]. Industries like copper smelters, iron and steel production, and municipal incinerators contribute to the pollution by copper [61]. Overall environmental copper pollution is well documented using the excellent attic dust approach, whereby dust collected from the attic of old houses is examined, providing an archive of historical air contamination by copper in the urban environment [71]. The observation of air contamination by copper and other heavy metals in the urban environment raised the question of potential health hazards [71], and as expected, there were similar discussions around this issue worldwide.

#### 3.2.2. Elucidating Health Hazards of Environmental Copper

While the existence of environmental copper pollution is well established, there is uncertainty about its effect on human health conditions because of divergent definitions of human health [72,73]. Classical medical research is disease focused and still defines health as the absence of disease [72], while languages associate a positive concept of wholeness with health as does the WHO health definition [73]. Newer medical health definitions emphasize the capacity to adapt to changing external and internal circumstances, and the results of the 2010 Global Burden of Disease study provides keys for quantifiable health metrics by developing statistical tools calculating healthy life expectancy as the sole defined end point [72]. As it currently stands, human health definitions remain vague because scored elements for a quantitative and qualitative evaluation outside of healthy life expectancy are not available.

Elucidating the environmental impact risk of copper on human health, including clinical liver injury, requires a careful and critical analysis of published proposals. Selected sources of relevant copper environmental pollution can be localized to several areas of interest and are briefly described, including critical comments (Table 2) [6,23,24,29,44,48,57,61,62,65,68,69,70,71,72,73,74,75,76,77,78,79,80,81,82,83,84,85,86,87,88,89,90,91].

It is interesting to see that the majority of the listed publications already have in the title wordings such as an assessment of health risks, environmental health hazards, or ecological health risk assessment (Table 2). In addition, the relationship of the potentially toxic copper with human health risks seems to be complex (Table 2). It is obvious that copper can be found as a contaminant in many places in the earth’s environment (Table 2).

Copper may enter the human body via gastrointestinal, pulmonal, or dermal uptake. The variable copper amounts leaving the soil or water contaminated with copper and finally arriving at exposed individuals could, theoretically, impair human health at various grades, but this indeed is not the case. In the clinical context, the amounts of copper derived from the environment and entering the human organs seem to be low, raising the question of their impact even at higher amounts on human health and the liver integrity. As expected, and considering the conclusions provided in all publications as listed (Table 2), firm evidence is lacking that contaminating copper may be injurious. Instead, contaminating copper is the best source supplying humans with this essential metal; it is natural and thereby free of costs and preferred to expensive commercial copper-containing supplements.

The mainstream opinion exists among theoretical toxicologists and clinical physicians that, in healthy individuals, perfect conditions of fine-tuned biological systems and mechanisms are available to ensure overall copper homeostasis not only within the liver but also in other organs [2,59]. Collectively speaking, if the uptake of copper is higher than the internal copper demand, excess copper will be excreted via bile or urine to sustain or obtain copper homeostasis, allowing for a healthy life [2,53,54,55,56,57,59]. These conditions apply on a day-by-day basis when humans are exposed to food, water, and air contaminated with copper, but they ultimately represent no health or liver risks that would need further fruitless time- and resource-consuming analytical efforts. 

The discussion of a possible health risk by contaminating copper now seemingly comes to a good end: not guilty. However, a cautionary statement is warranted if protective mechanisms providing copper homeostasis are not available in the few patients who suffer from genetic copper-storing diseases such as Wilson disease and Menke disease, or those experiencing intoxications by excess amounts of copper [92,93,94,95,96]. 

### 3.3. Acute Human Liver Injury by Exogenous Copper

Reports are lacking a description of acute liver injury in connection with exposure to environmental copper [25]. Acute copper poisoning is more often observed in South Asian countries where it is more prevalent in rural populations [92], while uncommon in Western countries [93]. Copper sulfate is easily available worldwide, and used in agriculture, the leather industry, and at home to make glue [92]. In addition, the burning of copper sulfate at home or in shops, seen as a good luck charm and also used for religious reasons, is a widespread practice in countries like India where Buddhists and Hindus live [92,94]. Copper intoxications have been reported worldwide following accidental or intentional exposure through various routes including oral [92,93,95,96] and inhalation [61]. Toxic amounts occur after the ingestion of as little as 1 g [93], while the lethal dose of ingested copper is 10 to 20 g [94]. About 60% of the ingested dose is absorbed in the gastrointestinal tract and attached mostly to ceruloplasmin (95%), whereas free copper binds to albumin, forming its toxic form [94,95,96,97].

The clinical manifestation of acute copper intoxication in one patient was substantial liver injury, with a serum AST of 2340 U/L and ALT of 780 U/L and a ratio of AST/ALT of 3; there were no ALP results with which to determine the R value to determine the liver injury pattern [93]. In another patient, serum ALT activity was 10 U/L with ALP activity of 406 U/L [94]. This was in line with a cholestatic liver injury as based on laboratory data [33]. Acute liver failure can develop due to direct copper toxicity [94,95,96,97]. In line with expectations, mechanistic steps leading to human liver injury due to acute intoxications by copper were not studied in acutely intoxicated patients [93,94,95,96,97]. However, it has been noted that copper toxicity is due to the generation of free oxygen radicals in the cells, causing severe intra-nuclear and cytoplasmic injury and cell death [96]. 

Clinical characteristics may include jaundice due to non-immune hemolysis as evidenced by a low blood hemoglobin level of 6.9 g/dL, low serum haptoglobin of <10 mg/dL, high serum activity of lactate dehydrogenase of 2148 U/L, increased serum unconjugated bilirubin of 4.5 mg/dL, and negative Coombs test [93], which classifies the hemolysis similar to the known hemolysis of genetic Wilson disease with a negative Coombs test [66], as opposed to autoimmune hemolysis with a positive Coombs test [98]. Copper can also lead to methemoglobinemia, in which the diagnosis of acute copper intoxication is confirmed by a total serum copper of 874 µg/dL and urine copper of 356 µg/24 h [93]. Intravascular hemolysis commonly starts as early as within the first 24 h of ingestion and is due to the direct oxidative damage to erythrocyte membranes [94]. The Cu^2+^ ion oxidizes the Fe^2+^ ion in hemoglobin to Fe^3+^ resulting in its conversion to methemoglobin [94,96]. This manifests as cyanosis and a loss of the oxygen carrying capacity of blood [94]. Copper causes direct injury to the proximal renal tubules and acute tubular necrosis with increased serum values of creatinine, with a maximum of 621 µmol/L as a sign of renal impairment, attributable also to dehydration, hemoglobinuria, rhabdomyolysis, and sepsis [94]. 

Evidence-based recommendations for the treatment of acute oral intoxications by copper are not available. This is due to the small numbers of affected patients that do not allow for valid randomized controlled trials (RCTs) evaluating efficacy and risks of therapeutic approaches. As a result, therapeutic measures are currently based on experiences as described in anecdotal reports. Gastric lavage is viewed as usually unnecessary due to persistent vomiting [93]. Particular care is certainly needed for spontaneous vomiting due to the risk of aspiration, especially in unconscious patients, who may require preventive intubation analogous to acute oral intoxications by aliphatic halogenated hydrocarbons like carbon tetrachloride or chloroform [20,21,22]. There is no place for intentional forced vomiting, and the use of activated charcoal was anecdotally reported despite a lack of efficacy [93]. The drinking of milk and physiological saline was occasionally recommended [94] but a better option may be the intestinal lavage to quickly remove the copper from the intestine, a procedure successfully applied in patients intoxicated by the ingestion of aliphatic halogenated hydrocarbons [20,21,22]. Chelating agents like D-penicillamine are commonly applied and recommended in severe poisoning, although pharmacokinetic data are scarce to guide their use [93]. Zinc occasionally was used [99]. The hemolysis-induced anemia is to be corrected by a transfusion of packed red blood cells [94,100], and hemodialysis may be indicated for renal insufficiency [94,100]. Renal failure will require hemodialysis to temporarily substitute the injured non-functional kidneys without the intention to remove the toxic copper from the blood [94]. Of note, some reports claim that through contaminated hemodialysis fluid infusions, the copper may be freed from the dialysis device and given to the dialyzed patients [92].

Prognosis is poor unless patients receive quick treatment with chelation and supportive measures in the face of lethality rates from 14% to 36% [92]. There is no supporting evidence that acute copper ingestion increases the risk of hepatocellular carcinoma. Most importantly, stopping the over-the-counter sale of copper sulfate and restricting the sale to authorized agents is strongly recommended to decrease the risk of acute copper intoxication, as is providing copper sulfate as large crystals, rather than as a fine powder easily dissolved [92,94]. 

### 3.4. Chronic Human Liver Injury by Exogenous Copper

In a patient with prolonged exogenous copper exposure for 10 years, the serum activity of ALT was 51 U/L, associated with an AST of 190 U/L and ALP of 68 U/L, but the absence of ULN data prevented a calculation of the R value [79]. The patient used up to 8 mg of copper as a daily dose for treating copper deficiency, known as human Swayback disease, whereby this iatrogenic copper overload led to liver transplantation due to compensated cirrhosis. LT values have not been reported in patients with acute renal failure following copper sulfate intoxication [101]. Similarly, no ALT or AST values are available as they were not analyzed in copper workers. 

In the patient with prolonged copper overdose, transjugular liver biopsy demonstrated, upon light microscopy, ongoing portal and segmental inflammation with a ballooning degeneration of the hepatocytes as well as diffuse hepatocellular copper accumulation on rhodamine [99]. The diagnosis of cirrhosis was first established at the occasion of a laparotomy for umbilical hernia repair as evidenced by macroscopical morphology and later confirmed at the time of liver transplantation, showing both micronodules and macronodules of the liver surface. An analysis of the hepatic content of copper provided extremely high values, suggesting copper as the causative agent in this patient [99]. Otherwise, convincing data of a direct mechanistic cascade of events leading to this liver injury due to a prolonged intake of exogenous copper are lacking but likely are similar to those described for Wilson disease, which is caused by genetic modifications, conditions that do not apply to the other forms of prolonged copper uptake. As a consequence, Wilson disease mimics chronic intoxication by copper only partially; the similarity is restricted to the liver injury and does not include the non-hepatic organs that are affected in Wilson disease due to local genetically impaired actions, circumstances that do not apply to non-Wilson diseases, such as in those chronically exposed to copper. 

Experimental studies in animals showed mostly unchanged, or in rare cases, slightly increased serum ALT and AST activities after the application of high copper amounts [102,103,104]. In animals, however, overdosed copper administration caused no liver injury as assessed by light microscopy [102,103,105] or only minimal, partially dose-dependent changes such as small vacuoles of hepatocytes, hepatocyte swelling, inflammatory cells, or sinusoidal congestion [104]. Electron microscopy data on the liver of the patient exposed to high amounts of copper were not available [99,101] but have been reported in animal studies as irregularly shaped nuclei, abundant mitochondria, and displayed cristae, and hepatocytes with an inclusion of secondary lysosomes [82]. Prolonged intoxication with exogenous copper has to be differentiated clinically from Wilson disease [66,105,106], a genetic disorder of the liver leading to hepatic copper accumulation [107,108]. 

The earlier termed Indian childhood cirrhosis (ICC) was attributed, in previous reports, preferentially to exogenous copper in drinking water or milk [109], which was obviously an assumption error [63,110,111,112]. In more detail, ICC was initially considered preventable and to be clearly distinguishable in Indian children from other chronic liver disorders including Wilson disease [109]. Grossly increased hepatic, urinary, and serum copper concentrations were described as characteristic findings of ICC. These increased concentrations were easily demonstrated histologically with orcein-rhodamine staining. The environmental ingestion of copper was the most plausible explanation for ICC, as shown by feeding histories, the prevention of ICC in siblings and in the Pune district by a change in feeding vessels, and the dramatic reduction in the incidence of ICC throughout India. The nature and role of a second factor in the causation of ICC remained unclear, although an inherited defect in copper metabolism was strongly suspected. ICC, however, was not considered to be a straightforward early onset of Wilson disease because ceruloplasmin was consistently normal and clinical and histologic recovery was maintained in the long term despite the withdrawal of D-penicillamine therapy. Descriptions of an ICC-like illness in the West suggested that different mechanisms including environmental and/or genetic factors can lead to the same end-stage liver disease: copper-associated childhood cirrhosis [111,113,114]. The early conclusion was reached that ICC probably represents a specific form of copper-associated childhood cirrhosis that requires high environmental copper ingestion for its full expression [109]. 

The initial concept of ICC [109] was challenged by subsequent studies [111,113]. While there is agreement that liver diseases of infancy and childhood are generally rare and within the spectrum of these disorders [109,113], only a few subtypes are related to abnormal hepatic copper accumulation [113]. In particular, idiopathic copper toxicosis has now been defined as such a subtype, characterized by distinct clinical and pathologic features; however, its exact etiology is still controversial [113]. Based on a review of the literature and observations of 138 cases endemic to western Austria, the hypothesis was presented that idiopathic copper toxicosis is caused by a constructive interaction of an autosomal-recessive inherited defect in copper metabolism and excess dietary copper [113], classified before as an ecogenetic disorder [112]. In line with these considerations is a case report from Germany on a female child of non-consanguineous, healthy German parents who fell ill at the age of 7 months with a progressive liver disease leading to irreversible hepatic failure 3 months later [111]. Histological examination revealed severe liver cell necrosis, excessive Mallory body formation and veno-occlusive-like changes associated with a massive storage of copper, similar to ICC. Chronic copper contamination of drinking water was the only detectable etiological factor. The conclusion is reached that ICC most probably is an environmental disease, also occurring outside the Indian subcontinent, and is likely to be underdiagnosed in the Western world [111]. 

This severe form of rapidly progressive cirrhosis associated with a marked increase in hepatic copper has been described in children from rural, middle class Hindu families in India [110,113]. Originally termed Indian childhood cirrhosis, similar clinical cases have been reported worldwide, and this disorder is now referred to as idiopathic childhood cirrhosis or idiopathic copper toxicosis [110,111,113,114,115]. Affected children are diagnosed by two years of age with hepatosplenomegaly, an elevation in the activity of serum aminotransferases, cirrhosis, and elevated liver copper [110]. Interestingly, the serum ceruloplasmin in these patients is normal or elevated, suggesting that the defect in biliary copper excretion is distal to the role of ATP7B in this process. Epidemiological investigations of idiopathic childhood cirrhosis indicate that both genetic and environmental factors may play a role in this disease [110]. Studies have revealed an increased copper content in the diet consumed by the affected children while an analysis of some families suggests autosomal recessive inheritance with incomplete penetrance [115]. In support of an underlying defect in hepatic copper excretion, D-penicillamine is effective in many cases and hepatic transplantation can be curative [110]. Overall, this disease, now called idiopathic childhood cirrhosis syn idiopathic copper toxicosis, is quite different from Wilson disease, traced back solely to genetic variability rather than to additional increased copper consumption through food or beverages.

### 3.5. Wilson Disease

#### 3.5.1. Natural Course

As a copper-storing liver disease caused by inheritable malfunctioning or missing ATP7B, Wilson disease is characterized by a disturbed cellular homeostasis of copper handling primarily in the liver cell [106]. Released from the liver cells due to limited storage capacity, the toxic copper enters the circulation and arrives at other organs, causing local accumulation and cell injury. This explains why copper injures not only the liver but is also responsible for liver-unrelated symptoms and various clinical features. 

#### 3.5.2. Clinical Characteristics

Wilson disease is a multifaceted disorder, difficult to diagnose and often misdiagnosed [106], in part due to physicians’ limited knowledge of its clinical features and a low prevalence, ranging from 1:40,000 to 1:50,000 in the general population [116]. In 22.5% of patients, the diagnosis was delayed and was not achieved until three years after the initial symptoms were evident, and the diagnosis was established at a mean age of 20.4 years (SD 10.6, range 4–56) [117]. However, Wilson disease has been described even in infants with an age range from a few months up to 4 years [118].

Apart from the liver, the most implicated organs were the brain, kidneys, eyes, heart, muscles, and bones [106]. Depending on the organ involved and the clinical stage of the disease, patients with Wilson disease may be polysymptomatic, oligosymptomatic, or monosymptomatic, but some patients may present even as asymptomatic, especially in initial stages of the disease. 

Details of clinical manifestations were perfectly and comprehensively listed in [106]: (1) findings related to the liver may include abnormal LTs, chronic active hepatitis, cirrhosis with portal hypertension, and acute liver failure; (2) psychiatric features comprise affect, cognition, and behavioral disorders as well as depression and psychosis; (3) neurologically, tremor, dysarthria, ataxia, nystagmus, writing problems, and dysphagia with pseudohypersalivation prevail; (4) renal tubular dysfunction; (5) Kayser–Fleischer corneal rings to be verified through split-beam examination by an eye specialist; and (6) various findings like cardiomyopathy, cardiac arrhythmias, rhabdomyolysis, osteoporosis, osteomalacia, arthritis, and arthralgia. In addition, Coombs-negative hemolytic anemia is a key feature of Wilson disease with undetectable serum haptoglobin, high serum activities of lactate dehydrogenase, and high reticulocyte counts [119,120]. 

#### 3.5.3. Diagnostic Approach

In any patient with suspected Wilson disease, first and second-degree relatives need to be screened for Wilson disease [2,121]. Such family screening facilitated the early diagnosis of Wilson disease, and substantially increased the prevalence, as the mean age of patients was significantly lower compared with patients diagnosed at a symptomatic stage of the disease (15.5 vs. 20.4 years; *p* = 0.021) [117].

A mutation analysis of the coding region of ATP7B (except exons two, three, and twenty-one) performed in 150 patients with Wilson disease showed no detectable mutations in 15% of patients, and mutations causing disease were found in 57% of patients on both chromosomes and in 29% of patients on one chromosome [122]. There were no significant differences in the frequency of pathological laboratory test values between the two study cohorts with detectable mutations. Thus, a negative ATP7B does not rule out Wilson disease, and the conclusion may be drawn that Wilson disease can develop without an association with ATP7B. Nevertheless, the search for ATP7B mutation is obligatory for a complete assessment in each patient with suspected Wilson disease [106] but certainly restricted in countries lacking test availability or confronted with high costs. 

There is not a single clinical feature viewed as key diagnostic sign that would help with the early recognition of Wilson disease [106,117]. It should be suspected preferentially in younger patients with jaundice as a sign of liver disease or hemolysis, or if psychiatric or neurologic symptoms are evident [106,117]. In infants before the age of three years Wilson disease is uncommon [60]. As expected, liver injury as one of the key features in Wilson disease received much attention in various publications [115,116,117,118,119,120,121,122]. Among these, liver manifestations were recently summarized [122]. Accordingly, patients may be asymptomatic, especially in the early disease stage, whereas symptoms may start with fatigue, reduced appetite, and eventually jaundice. With the development of jaundice, patients present to their practitioner, who may establish or even miss the diagnosis of Wilson disease. Provided hemolysis is excluded, jaundice is commonly due to acute flares of any hepatobiliary disease including Wilson disease. In Wilson disease, jaundice may reflect acute liver injury with a sharp elevation of LTs. Later stages may include hepatomegaly as well as fibrotic or cirrhotic changes of the liver, as evidenced by portal hypertension with splenomegaly, ascites or bleeding esophageal varices as signs of decompensated cirrhosis [122]. In addition, 3–5% of the Wilson patients present with acute liver failure requiring urgent liver transplantation [122].

Laboratory results of serum LTs are variable and not helpful to suspect the diagnosis of Wilson disease. Serum ALP activities were described with low levels for unknown reasons [2,123,124]. In Wilson disease serum activities of AST are often much higher as compared with ALT [119,123]. High serum ALT activities up to 800 U/L were commonly found only in younger children with an age range from 4 to 8 years, with low ALT activities in infants with an age <4 years partially due to hemolytic crisis, and higher ALT values in 3 other infants [118]. Consequently, a realistic R value to determine the liver injury pattern based on LT values is not feasible, as also seen in a recent study providing serum activities for ALT of 46.9 ± 33.8 U/L and for ALP of 158.0 ± 119.4 U/L but without giving ranges of normal [124].

Moreover, increased plasma levels of inflammatory cytokines and chemokines were found in patients with Wilson disease as compared with healthy controls, an interesting finding but not contributing as a diagnostic biomarker [125]. Serum autoimmune parameter including anti-nuclear antibodies (ANA) were rarely reported at the start of the therapy, with higher frequencies during treatment with D-penicillamine [126]. Other laboratory parameters in patients with Wilson disease were abnormal, including serum ceruloplasmin <0.2 g/L, non-ceruloplasmin-bound serum copper >25 µg/L, urinary copper excretion >1.6 µmol/24 h, and liver copper >250 µg/g dry wt [122].

Mainstream opinion describes Wilson disease as a disorder difficult to establish as a firm diagnosis due to variable clinical features and abundant laboratory results that differ in their validity [106,122]. In this context, artificial intelligence (AI) provides a forum whereby complicated processes including those prevalent in the diagnosis of human diseases are solved by intelligent diagnostic algorithms using relevant characteristics that summarize given individual scores, providing a final score with grades of probability [41]. Using such a scoring method, complex diagnoses of various diseases were firmly diagnosed, including DILI and HILI [32,33,41,42], autoimmune hepatitis [127], and Wilson disease, for which the Leipzig scoring system of 2003 [128] or the subsequently modified Leipzig soring method of 2019 was successfully applied [129]. Key items of Wilson disease received individual scores, and the sum of these scores classified the suspected Wilson disease as an established diagnosis (score ≥ 4), as possible (score 3), or as very unlikely (score ≤ 2) (Table 3). 

This modified Leipzig score was derived from a previous report [129], but for reasons of precision, the score was now revised: under the item of 24 h urinary copper, the previous term “acute hepatitis” was replaced by “chronic cholestatic liver disease”. 

The note in the modified Leipzig scoring system as published [129] under the item 24 h urinary copper (in the absence of acute hepatitis) is not based on any evidence as plain acute hepatitis is not known for increased urinary copper excretion, as opposed to cholestatic autoimmune hepatitis (AIH) or any other liver disease presenting with cholestatic features like primary biliary cholangitis (PBC) or primary sclerosing cholangitis (PSC) [66,130], which may masquerade as Wilson disease due to high urinary copper excretion [130]. For all three cholestatic liver diseases (AIH, PBC, and PSC), a range of diagnostic parameters are available for exclusion purposes (Table 2). Thus, the term acute hepatitis has now been replaced by chronic cholestatic liver disease (Table 3). 

Liver histopathology shows, in the initial stages of Wilson disease, mild unspecific lobular changes, and occasionally also shows singular apoptotic hepatocytes and spotty necrosis with surrounding lymphocytes, mild macrovesicular steatosis, and ballooned or glycogenated hepatocytes [131]. With progressing disease, inflammation increases, leading to fibrosis und ultimately to cirrhosis with the clinical end stage of acute liver failure. Increased copper accumulation in the hepatocytes may be visible by histochemical staining with rhodamine or rubeanic acid through direct binding to copper [131,132]. However, the absence of visible copper-binding protein does not exclude Wilson disease [131]. More specifically, at the time of diagnosis, liver histology obtained by liver biopsy in 78 patients showed chronic liver injury in 73% of patients, with fibrosis occurring in 36% and cirrhosis in 37% of patients, while steatosis was found in 54% of the evaluated liver specimens and a normal liver histology was described in three patients [122].

Ultrastructural analysis by an electron microscopy of the liver at the early Wilson disease stage of steatosis reveals specific mitochondrial abnormalities [133,134]. Typical findings include variability in size and shape, increased density of the matrix material, and numerous inclusions including lipid and fine granular material that may be copper. The most striking alteration is increased intracristal space with a dilatation of the tips of the cristae, creating a cystic appearance [134]. In the absence of cholestasis, these changes are considered to be pathognomonic of Wilson disease. At later stages of the disease, dense deposits within lysosomes are present. Ultrastructural analysis may be a useful adjunct for diagnosis [133].

#### 3.5.4. Therapy

There are no prospective studies using RCTs available with a focus on the efficacy and risks of therapeutic approaches in patients with Wilson disease [66], partly attributable to the low disease frequency [129,135]. However, as for any new therapy approach, a control group would necessarily have any drug treatment withheld, but this would provide them with treatment too late and would be unethical. Instead, empirical therapy was applied early based on anecdotal reports, personal experience, and considering mechanisms leading to the liver injury [66,133]. To achieve a reduction in copper excess in the liver, two strategies have to be discussed, one refers to the uncommonly proposed prevention through a reduction in alimentary copper [52,57,66,135], and the other one focuses on increasing renal copper elimination as the better alternative [66,120]. 

Most of the commonly consumed foods contain trace amounts of copper [66,136], which makes dietary copper restriction less feasible, although foods with high copper content should be avoided to support therapy with chelators [66]. Foods with a high copper content include cocoa derived from Owena cocoa (Theobroma cacao L.) and contaminated with copper from copper-containing fungicides, dark chocolate from contaminated cocoa, nuts, raisins, shellfish, oysters, and butchery foodstuff from liver and kidneys derived from cattle grazing, possibly, on grounds with plants contaminated by copper [67,68,128,136].

Patients with Wilson disease are well treated first line with copper chelators, like D-penicillamine, that help remove circulating copper bound to albumin, which facilitates urinary copper excretion via the kidneys [66]. Based on previous considerations that drug treatment with D-penicillamine in patients with Wilson disease may interfere with pyridoxin, comedication with pyridoxal 5′phosphate, the biologically active form of vitamin B6 25–50 mg daily is commonly prescribed [106]. However, a recent preliminary study from France questioned the need for a routine comedication with vitamin B6 as its blood levels were in the normal range in Wilson disease patients treated with D-penicillamine in the absence of additional vitamin B6 supplementation [124]. In this context, it was mentioned that the usual diet contains sufficient amounts of vitamin B6, and vitamin B6 needs to be supplemented only if food deficient in vitamin B6 is consumed. There was also the note that optic neuropathy was observed in four patients in association with a D-penicillamine therapy [124]. However, a temporal association is not necessarily a causal association. Finally, it was argued that little published data on vitamin B6 supplementation for patients treated with D-penicillamine is available and no consensus recommendation exists [124].

D-penicillamine is initially given at a daily dose of 300 mg and increased weekly by 300 mg up to 1500 mg/d [66]. Remission is achieved with the normalization of free copper blood levels and urinary copper output, but lifelong maintenance treatment is mandatory with mostly 600–900 mg daily [128]. Similar to any other drug treatment for most diseases, the therapy with D-penicillamine is not without adverse drug reactions (ADRs). Initial allergic reactions can be scoped by intermittent drug cessation or concomitant use of corticosteroids [66,117,124]. More serious is an initial deterioration of neurological symptoms occurring in 14% of treated patients, hardly to be reversed and of unknown etiology [66,117]. Of concern is the development of autoimmunity due to D-penicillamine use, as evidenced by a new appearance of serum anti-nuclear antibodies (ANA) with variable titers [128] and to be differentiated from increased ANA titers already occasionally found before starting drug therapy for Wilson disease itself [126]. In the case of new autoimmunity, switching from D-penicillamine to alternative, second-line therapies of zinc is recommended [66].

For a long time, treatment with trientine has been recommended as a second-line therapy for patients with Wilson disease although no studies with head-to-head comparisons for initial treatment were available [66,133,137,138,139]. Respective previous recommendations are now outdated. Therapy with trientine 2HCl was restricted to patients intolerant to D-penicillamine and requires slowly increasing doses up to 1800 mg daily and reducing the doses down to 600–1000 mg daily, sufficient as maintenance doses for most patients [66,137]. Comedication with B6 was not required. Treatment efficacy was previously found to be similar for D-penicillamine and trientine, with fewer ADRs for trientine although neurologic features may worsen [66,133]. Also on the market are trientine 4HCl, which is stable at room temperature, and trientine 2HCl, which requires storage at 2–8 °C [138]. As copper-chelating agents, the trientine salts bind with excess copper, forming a stable complex which is excreted mainly in the urine [138]. It has been suggested that trientine salts may also decrease intestinal copper absorption [133,138,139].

The use of zinc as zinc salts or the more tolerable zinc–amino acid-bound medications, is associated with fewer ADRs except for severe abdominal discomfort that may lead to drug cessation [66]. The recommended dose for adults is 3 × 50 mg daily and for children or adolescents 3 × 25 mg daily [140]. Regarding the amelioration of liver injury, it was deemed inferior to chelators [66,135,137,141]. Zinc induces intestinal metallothionein, which blocks copper absorption, increases excretion in the stools, and results in an improvement in symptoms [141]. In addition, two meta-analyses and several large retrospective studies indicate that zinc is equally effective as a chelator for the treatment of Wilson disease, with the advantages of a very low level of toxicity and only the minor side effect of gastric disturbance. Thus, zinc may have a role as a first-line therapy in Wilson disease patients with neurological symptoms [133,141], is gaining acceptance for patients with hepatic presentations, and is universally recommended for lifelong therapy [141].

A short note on Bis-choline tetrathiomolybdate (TTM), also known as ALXN1840, is warranted as it is a drug in the pipeline of AstraZeneca for treating Wilson disease. TTM was recently cut at clinical phase III after results of additional phase II studies were disappointing, not reaching the study aims, and following consultations with regulatory agencies, communicated in spring 2023 by Pascal Soriot as the Chief Executive Officer (CEO) of AstraZeneca [142]. On theoretical grounds, treatment with TTM appeared promising due to its capacity to increase biliary copper excretion [66,142,143,144,145]. However, little attention was paid to the fact that copper may be reabsorbed from the intestine via the entero-hepatic circulation, counteracting hepatic copper depletion. Moreover, any new therapy approach should be more effective compared with previous treatment modalities like D-penicillamine, trientine, or zinc. Although initial studies with TTM were viewed as positive [66,143,144,145], some criticism was communicated regarding the low case numbers in the study cohort and quantitative copper measurement for assessing efficient copper removal from the blood by an old drug in a new design for Wilson disease, raising the question of whether TTM is good for the brain and liver [144]. It will be interesting to see the final information from AstraZeneca on why clinical phase III was cut [110]. 

Liver transplantation is an option as an ultima ratio in the end stage of Wilson disease, including in cirrhosis with untreatable complications or acute liver failure, providing a life-saving approach, curative treatment of the disease, restoration of the liver function, and mitigation of portal hypertension [146,147], with excellent survival rates of one and five years without disease recurrence [146]. In the case of deceased donor liver shortage, living related liver transplantation is an option [146]. Although promising data were obtained in animal studies, future RCT studies in humans are required to assess innovative approaches directed at hepatocyte transplantation [146,148], the transplantation of bone marrow cells [149], and gene therapy [106,146]. The indication for a liver transplantation has to be carefully considered in Wilson disease patients with severe psycho-neurological symptoms [146,147]. 

#### 3.5.5. Prognosis

Prognosis is poor in patients with Wilson disease without the benefit of drug therapy, with the median life expectancy reduced to 40 years [66,106]. Instead, the prognosis is commonly good in Wilson disease, provided the disease is diagnosed early and patients receive an appropriate drug therapy, which might, rarely, include liver transplantation [66]. Under these conditions, the cumulative survival in 51 patients with Wilson disease was 95% at 33 years after diagnosis and identical with 95% of a control group matched for sex and age, considering that survival was only slightly reduced during the early observation period when liver transplantation was not available for acute liver failure.

During the observation period of 33 years and under the D-penicillamine treatment, all clinical signs improved with the exception of gynecomastia and esophageal varices, and treatment was associated with the improvement of all neurological symptoms and amelioration of all hematological laboratory test results [66,106]. 

#### 3.5.6. Cascade of Molecular Events Leading to Copper Liver Injury

A broad range of proposals on how copper may cause molecular and mechanistic liver injury have been published [25,26,150], based on human or animal data [99]. Mechanistic events are similar in animals or humans exposed to high amounts of exogenous copper [92,99] compared to patients with Wilson disease, except for their genetic ATP7B mutations as the basis for this specific clinical liver injury [2,66] and transcuprein/alpha-2-macroglobulin of the blood following intestinal uptake, regulated by demand through intestinal transporters [151].

The second step represents the genetic impairment of biliary copper excretion [133]. This causes copper overload not only in the liver but also in other organs [66].

Third, the oxidized form of copper is the injurious metal that first attacks subcellular organelles of the hepatocyte like mitochondria, as evidenced by electron microscopy in patients with initial stages of Wilson disease [133,134]. These ultrastructural mitochondrial injuries are associated with functional disturbances of the mitochondrial respiratory chain, responsible for energy by ATP production and fatty acid oxidation [152,153], that cause steatosis, visible with light and electron microscopy [131,133,134]. Apart from liver mitochondria, other organelles of the liver cells can be damaged, because their membranes contain proteins and unsaturated fatty acids, which can easily be attacked by reactive oxygen species (ROS) [154] and generate lipid peroxides as evidenced by high plasma malondialdehyde (MDA), low glutathione (GSH) levels, and reduced total antioxidant capacity (TAC) [155,156]. Toxic intermediates are generated in the course of an interaction between the reduced form of copper and oxygen via the Fenton reaction [154], leading to superoxide anion, hydrogen peroxide, and hydroxyl radicals comprising ROS generated through oxidative stress, and capable of triggering, at least partially, liver injury [99,150]. In this context, cuproptosis is a newly described copper-dependent form of regulated cell death considered to be a contributory causative mechanism in Wilson disease [150]. Copper induces the aggregation of lipoylated dihydrolipoamide S-acetyltransferase (DLAT). This is associated with the mitochondrial tricarboxylic acid (TCA) cycle, resulting in proteotoxic stress in line with the proposed cuproptosis [150]. The copper-dependent, regulated cell death is distinct from known death mechanisms because of its dependency on the mitochondrial respiration. Notably, copper hepatotoxicity is not just oxidative stress by this heavy metal because zinc, by replacing copper, may be a major cofactor in various cellular processes of liver injury [157]. 

Fourth, recent evidence suggests a correlation between dysbiosis in gut microbiome and multiple diseases such as genetic disorders, including Wilson disease. As an example, 16S rRNA sequencing was performed on fecal samples from 14 patients with Wilson disease and was compared to the results from 16 healthy individuals [158]. The diversity and composition of the gut microbiome in the Wilson disease group were significantly lower than those in healthy individuals. The Wilson disease group presented a unique richness of *Gemellaceae*, *Pseudomonadaceae*, and *Spirochaetaceae* at the family level, which was hardly detected in healthy controls. This group had a markedly lower abundance of *Actinobacteria*, *Firmicutes*, and *Verrucomicrobia*, and a higher abundance of *Bacteroidetes*, *Proteobacteria*, *Cyanobacteria*, and *Fusobacteria* than healthy individuals. The Firmicutes to Bacteroidetes ratio in the Wilson disease group was significantly lower than that of healthy controls. In addition, the functional profile of the gut microbiome from Wilson disease patients showed a lower abundance of bacterial groups involved in the host immune- and metabolism-associated systems pathways such as transcription factors and ABC-type transporters, compared to healthy individuals. These results implied the dysbiosis of gut microbiota may be influenced by the host metabolic disorders of Wilson disease, which may provide a new understanding of the pathogenesis and even new possible therapeutic targets for Wilson disease. However, the impact of intestinal microbiota polymorphisms in Wilson disease have not been fully elaborated and need to be explored for seeking some microbiota benefit for Wilson disease patients [158].

As the fifth step in the further course of Wilson disease, hepatic immune cells become more involved, as evidenced by the detection of inflammatory mediators like cytokines in the plasma of patients with Wilson disease, changing a silent liver into an organ that provides information on processes occurring in the injured human liver to the blood of patients, easily available for further analysis by physicians [154]. The interaction of copper with immune cells producing mediators is well documented [159,160,161], explicitly shown by the reduced interleukin-2 (IL-2) production and IL-2 mRNA in human T lymphocytes caused by copper deficiency [161]. In this context and of importance regarding mechanistic steps in Wilson disease, there are results of an increased expression of cytokines and chemokines in the plasma of patients, indicating that the dysregulation of mediators plays a role in Wilson disease [154]. The study cohorts consisted of 99 patients with Wilson disease and 32 healthy controls. Compared with controls, in patients with Wilson disease, there was a significant increase in plasma of T helper (Th) 1 cells (IL-2, TNF-α, and TNF-β), Th2 cells (IL-5, IL-10, and IL-13), and Th17 (IL-23) (*p* < 0.05). Higher plasma Th 1 cells (IL-2, TNF-α, and TNF-β), Th 2 cells (IL-13), and Th 17 (TGF-β1, IL-23) levels were found in neurological patients compared with control groups (*p* < 0.01). In addition, Th 1 cells (TNF-α and TNF-β), Th 3 (TGF-β1), and Th 17 (IL-23) levels were significantly higher in hepatic and neurological patients (*p* < 0.05), whereby the higher Th1 cells (IL-2, TNF-α, and TNF-β), Th2 cells (IL-13), and Th17 (TGF-β1, IL-23) and the course of Wilson disease were associated with the severity of the neurological symptoms for Wilson disease patients [154]. Finally, a prolonged exposure of the liver to considerable amounts of copper will activate resident hepatic stellate cells to myofibroblasts, which secrete extracellular matrix proteins that generate collagen [162], leading to liver fibrosis and cirrhosis [106,122]. Despite some uncertainties, it remains to be established whether animal models [163,164], including the goldfish model [105] or the zebrafish model [165], can contribute to close the existing mechanistic gaps of excess copper liver injury. 

Critical issues related to the liver injury due to copper have been discussed and quoted [99,150] and are now summarized (Figure 3).

## 4. Iron

Iron (Fe, from Latin: ferrum) is environmentally present [1,2,3,4,5] and became an indispensable element in humans for several vital biological processes [166] and in many other living organisms [167] including plants and microorganisms [168]. In a physiological range, iron is beneficial for human health, but lower or higher amounts are risky. Like other living organisms, humans cannot synthesize the metal but must acquire it by food from plants and the meat of cattle that consume plants rich in iron [166].

### 4.1. Physiology

As opposed to other minerals, iron levels in the human body are only controlled by absorption [166,169,170]. In line with experimental studies in rats [171,172], iron uptake in humans proceeds in the upper small intestine by an active, carrier-mediated mechanism involving a membrane iron-binding glycoprotein [170]. Intestinal absorption largely ensures body iron homeostasis in humans because iron lacks active excretory mechanisms [169,173]. 

Dietary iron is taken up by the divalent metal transporter 1 (DMT1) in enterocytes and moved to portal blood via ferroportin (FPN), where it is bound to transferrin and taken up by hepatocytes, macrophages and bone marrow cells via transferrin receptor 1 (TfR1) [167] with hepatic macrophages playing a crucial role for iron homeostasis [174]. As an unregulated process, iron excretion occurs through loss in sweat, menstruation, and the shedding of hair and skin cells, associated with rapid turnover and the excretion of enterocytes. 

**Figure 4:**
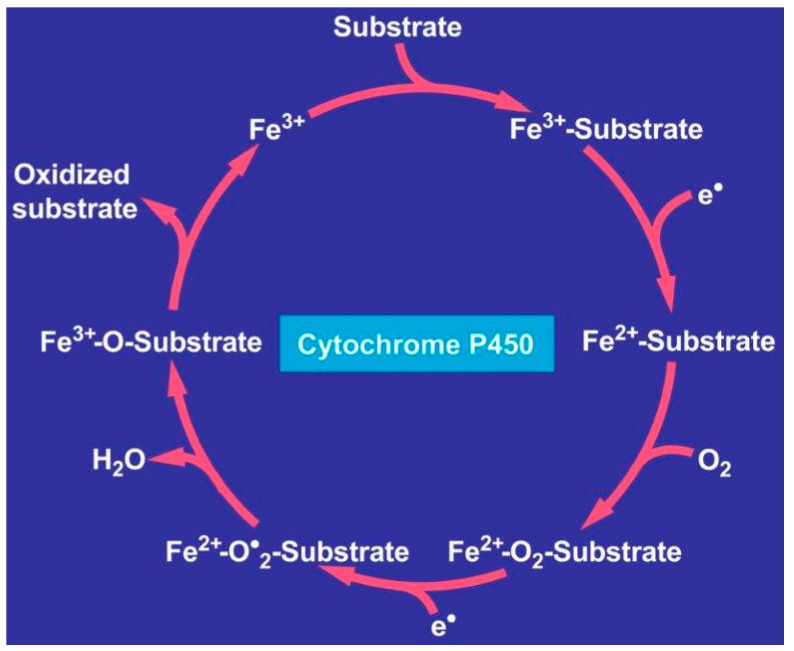
Catalytic CYP cycle. Hepatic microsomal cytochrome P450 requires iron and releases it upon its degradation. Abbreviations: CYP, cytochrome P450; ROS, reactive oxygen species. The figure is derived from an open access report [18].

In the human body, iron exists mainly in erythrocytes as the heme compound hemoglobin with approximately 2 g of iron in men and 1.5 g in women, to a lesser extent in storage compounds like ferritin and hemosiderin, and in muscle cells as myoglobin [166]. Iron participates in ferroptosis [175,176] and DNA synthesis, electron transport, and oxygen transport [166]. The metal is also bound to proteins such as hemoproteins and non-heme enzymes [167] involved in oxidation–reduction reactions and the transfer of electrons (cytochromes and catalase) [166], with iron embedded in cytochrome P450 (CYP) [177] required for the metabolism of drugs and other exogenous substrates shown for the catalytic CYP cycle (Figure 4) [18]. 

Under physiological conditions, iron in humans is commonly bound to hemoglobin in erythrocytes, while iron from senescent red blood cells is recycled by macrophages in the spleen, liver, and bone marrow [167]. Whereas most of the physiologically active iron is bound to hemoglobin, the major storage of iron occurs in the liver in a ferritin-bound fashion. In response to an increased iron load, hepatocytes secrete the peptide hormone hepcidin, which binds to and induces the internalization and degradation of the iron transporter ferroportin (FPN), thus controlling the amount of iron released from the cells into the blood. 

In the blood, iron is commonly bound to transferrin as transferrin-bound iron (TBI). However, a small pool is also present as NTBI [167,178] as the major contributor to the iron loading of hepatocytes when transferrin is saturated [179]. In addition, liver hepatocytes ensures iron homeostasis by producing and releasing the 25-amino acid peptide hormone hepcidin [178]. Hepcidin is secreted into the bloodstream and inhibits the release of iron in several cells, such as duodenal enterocytes, macrophages, hepatocytes, and Kupffer cells [180]. By binding to FPN, hepcidin facilitates the ubiquitination, internalization, and degradation of FPN as well as directly blocking the channel for iron export out of the cell into the plasma [167,181]. The synthesis of the peptide hormone FPN is regulated at the transcriptional level, and controlled by serum iron concentrations [182]. When serum iron levels are increased, hepcidin expression is upregulated and results in the blocking of iron transport to the plasma via FPN, thus providing a negative feedback response preventing potential toxic iron accumulation in the body [167,182]. Low plasma levels of iron lead to low transferrin saturation [183], which in turn impairs the synthesis of hepcidin [182]. Therefore, iron concentration in biological fluids is tightly controlled to provide adequate intracellular and extracellular iron levels, thus preventing its toxic accumulation [183]. This is a key process, as any abnormalities in the distribution and content of iron in the body can have harmful effects on the physiological processes through ferroptosis, a new form of programmed cell death, which develops with iron dependence and occurs in patients with hemochromatosis [167,176]. 

The laboratory assessment of the iron status relies on serum parameters such as serum ferritin (SF), transferrin saturation, and soluble transferrin receptor (sTfR) [184]. These indicators present challenges for clinical practice and national nutrition surveys, and iron status interpretation is often based on the combination of several indicators. The diagnosis of iron deficiency (ID) through SF concentration, the most commonly used indicator, is complicated by concomitant inflammation since SF is also a diagnostic biomarker of acute phase inflammation [184]. On the other hand, sTfR concentration is an indicator of functional ID that is not an acute-phase reactant, but challenges in its interpretation arise because of a lack of assay standardization, common reference ranges, and common cutoffs [184]. It is unclear which indicators are best suited to assess excess iron status. The value of hepcidin, non-transferrin-bound iron, and reticulocyte indexes is being explored in research settings. Serum-based indicators are measured on fully automated clinical analyzers available in most hospitals. Although international reference materials have been available for years, the standardization of immunoassays is complicated by the heterogeneity of antibodies used and the absence of physicochemical reference methods to establish “true” concentrations. From 1988 to 2006, the assessment of iron status in NHANES was based on the multi-indicator ferritin model. However, the model did not indicate the severity of ID and produced categorical estimates. More recently, iron status assessment in the National Health and Nutrition Examination Survey (NHANES) has used the total body iron stores (TBI) model, in which the log ratio of sTfR to SF is assessed. Together, sTfR and SF concentrations cover the full range of iron status [184].

Serum parameters of normal and pathological iron status may differ among various laboratories depending on the analytical method used and definition of a cutoff, and are highly variable, influenced by age and gender ranges of normal [184,185]. While the determination of serum iron is of no clinical relevance due to its variability and lack of specificity, among the most used parameters in evaluating iron status are serum ferritin in combination with fasting transferrin saturation, although ferritin is also an acute phase protein that is increased in inflammatory conditions, confounding the interpretation of obtained values [185]. 

Iron as a trace metal in physiological amounts is beneficial [166,167] due to excellent measures that provide iron homeostasis, as comprehensively reviewed [167]. However, iron deficiency may be hazardous to human health due to anemia and various clinical manifestations [186], easily seen externally by pale skin and cheilitis [187,188]. Iron may also be injurious when present in excess due to acute and chronic intoxication by exogenous iron or in the course of genetic hemochromatosis with a hepatic overload of iron due to uncontrolled intestinal iron uptake [66,169,170,171,172], which is to be clinically differentiated from hemosiderosis with iron excess in the hepatic Kupffer cells due to a chronic release of iron from injured blood red cells as a consequence of genetic hemolytic disorders [189,190].

### 4.2. Iron as Pollutant and the Issue of Health Hazards

#### 4.2.1. Sources of Iron as an Environmental Pollutant

Iron is the second most abundant metal in the earth’s crust, of which it accounts for about 5%, and is commonly found in nature in the form of its oxides [191]. The presence of iron was documented in different environmental matrices such as air, water, soil, food, plants, animals, and humans. This metal is one of the most common pollutants present in drinking water [191] but it was not found in plants of a municipal solid waste landfill [23]. Ethnomedical studies on the iron content of some medicinal herbs used in traditional medicine in Cote d’Ivoire for the treatment of anemia were reported [192], and iron as a suspected hepatotoxic pollutant was described in relation to medicinal herbs but studies on liver injury in patients using these herbal medicines are not published [24]. 

Environmental iron pollution is reported in the attic dust study, which used dust collected from the attic of old houses as an archive of historical air contamination by iron in the urban environment [71].

#### 4.2.2. Elucidating Health Hazards of Environmental Iron

Several reports presented details and proposals for potential health hazards in connection with exposure to environmental iron. They were provided in condensed form and supplemented by critical comments if needed (Table 4) [183,193,194,195,196,197,198,199,200].

Overall, published work has revealed little if any convincing evidence of potential health hazards during and after exposure to iron derived from the environment (Table 4) [183,193,194,195,196,197,198,199,200]. Assessing the various key aspects is certainly a challenging approach as many confounding variables must be considered. They include different oxidation forms of the iron ion with different solubility characteristics. In principle, the environmental iron occurs in two stable oxidation states, preferentially as ferric (Fe^3+^) and ferrous (Fe^2+^), yet ferrous iron (Fe^2+^) is the form that is transported into enterocytes [201,202,203,204]. The oxidized or ferric (Fe^3+^) form is highly insoluble and must, therefore, be reduced to absorbable ferrous before transport through the intestinal mucosa. After entering the human gastrointestinal tract, the oxidized iron is poorly absorbed, conditions that help reduce the intestinal uptake of iron in amounts higher than needed for iron homeostasis. Many proposals were only based on theoretical considerations, like the hazard index, without proof that the iron actually entered the body of the exposed humans, because amounts of iron were not quantified [19,193,194,195,196,197,198,199,200]. There is also an information gap on how the highly insoluble ferric is absorbed by the pulmonary mucosa after exposure to the lungs by inhalation. Open for discussion is the assumption that neurogenerative diseases are causally related to the uptake of environmental iron (Table 4), especially in view of the commonly accepted view that demethylation in the basal ganglia is closely associated with age-related iron accumulation, though the pathophysiological mechanisms of the local iron accumulation have not yet been studied [205]. 

In essence, overall potential health risks following exposure to environmental iron lack the required level of evidence and seemingly are negligible, although some authors attributed a few disorders to the action of environmental iron (Table 4). However, healthy individuals not affected by gene mutation, which may disturb cellular iron homeostasis, are well prepared to manage increased amounts of iron entering the body through a reduction in intestinal iron uptake. Of course, this special approach of reduction does not work in patients with genetic hemochromatosis and in individuals exposed to iron in a setting of intoxication.

### 4.3. Acute human Liver Injury by Exogenous Iron

Acute intoxications with iron commonly occur through ingestion by intention or unintentionally [206,207,208,209,210,211,212,213,214,215]. There are reports on fatal iron toxicity in adults causing fulminant hepatic failure [208,212,213] with normal serum iron levels at 36 h after ingestion due to the redistribution of iron to the intracellular compartment [212]. In another patient, plasma iron levels were high on day 3 after intoxication with a further increase on day 6 and a concomitant decrease in unbound iron binding capacity, whereas plasma ferritin levels were high on day 3 [208]. In this patient, a radiodense content in the stomach was described on day 1, valuable information for future cases, and plasma activities were increased on day 2 with ALT and AST > 15,000 U/L, followed by a rapid decline to normal values. Despite repeated gastric and intestinal lavages, chelating therapy, and plasma exchange, the patient died of fulminant hepatic failure, and upon autopsy, liver histology showed the complete disappearance of hepatocytes [208]. There are no evidence-based guidelines on how best to treat patients with acute iron ingestion. Occasionally, a tightly packed collection of iron known as a bezoar is detected in the stomach if substantial amounts were ingested, for which endoscopic removal should first be approached before operative removal is considered [214]. 

Unintentional iron ingestion is one of the most common deadly poisonings among children through drugs containing iron prescribed to patients with anemia [209]. In this cohort, clinical features of acute iron ingestion are similar to those in adults [208]. Overall, details of cascade events leading to liver injury by acute iron intoxications in individuals lacking genetic hemochromatosis characteristics are, as expected, not available but likely are similar to those described below for hemochromatosis.

### 4.4. Chronic Human Liver Injury by Exogenous Iron

In a recent case report on prolonged iron use leading to hepatic overload, a postmenopausal patient consumed, for 30 years after menopause, 1–3 tablets daily, each containing 325 mg ferrous sulfate, because of a mistaken belief that it would benefit her general health [216]. Her serum transaminases and ALP activities were normal, but serum ferritin level was strikingly increased. In addition, magnetic resonance imaging (MRI) of the abdomen showed iron deposition in the liver and spleen compatible with overload by exogenous iron. In the liver histology, increased iron was found in Kupffer cells and hepatocytes with focal fibrosis but no cirrhosis. The issue of mechanistic steps involved in this type of prolonged human liver injury by iron attracted little scientific interest and led to no published data. However, respective pathogenetic events are likely similar to those of hemochromatosis with its characteristic features outlined further below. Both disease entities partially show a similar involvement of the liver, although the risk of liver injury is higher in the genetically based hemochromatosis. Studies on other similarities comparing both disease entities were not published. 

Chronic exposure to iron oxide in steelworkers was reported in an early study but liver enzymes were not analyzed because the focus was on pulmonary aspects [217], confirmed by a later report with a focus on chronic obstructive pulmonary disease (COPD) [218]. A critical reappraisal report concluded that prolonged overload from exogenous iron rarely causes serious liver injury in humans and is only weakly fibrogenic in animal models, calling into question the concept that iron overload is a compelling cause of hepatotoxicity [219], in line with recent views [25,26]. 

### 4.5. Hemochromatosis

Hemochromatosis occurs in homozygotes with a mutation of the hemochromatosis gene (HFE) protein with a prevalence of 1:300 to 1:500 individuals [189]. A mutation in the HFE gene causes an increased absorption of iron despite a normal dietary iron intake. C282Y and H63D are the most common mutations of the HFE gene, present on the short arm of chromosome 6 (6p21.3) [189,220]. Different types of hemochromatosis are currently under discussion [189,221]; the most recent proposals are as follows [189]:

- Type 1 (HFE-related): This is the classic form of hemochromatosis that is inherited in an autosomal recessive fashion with worldwide prevalence [222,223].
- Type 2a (mutations of hemojuvelin gene) and type 2b (mutations of the hepcidin gene): Autosomal recessive disorder that is seen both in whites and non-whites. Its onset is usually at 15–20 years [189].
- Type 3 (mutations of transferrin receptor-2 gene): Autosomal recessive disorder that is seen both in whites and non-whites. Its onset is at 30–40 years [224].
- Type 4 (mutations of the ferroportin gene): Autosomal dominant disease that is seen both in whites and non-whites. Its onset is at 10–80 years [189].

Hemochromatosis, with its retained iron primarily deposited in the hepatocytes, is to be differentiated from hepatic iron overload syn hemosiderosis with its iron primarily deposited as hemosiderin in the reticuloendothelial cells of the liver [189]. This form of hepatic iron overload can occur in a variety of conditions like anemia with ineffective erythropoiesis due to thalassemia, sickle cell anemia, and hereditary spherocytosis, overall conditions often associated with multiple transfusions of red blood cells to treat anemia [190]. If iron overload is severe, chelation as a first-line therapy is indicated to avoid disease progress. Of course, venesection is not possible due to already existing anemia. 

#### 4.5.1. Natural Course

Hemochromatosis is a genetically heterogeneous disorder [189,221] that causes iron overload by an excess intestinal absorption of dietary iron, due to a decreased expression of intestinal hepcidin [225,226]. Excess amounts of iron are primarily found in the liver with its hepatocytes, which comprise around 80% of liver mass and are able to synthesize a high amount of the iron storage protein ferritin [167]. Iron is also stored in other parenchymal cells like pancreatic cells and cardiomyocytes [227] as well as joints, thyroid, skin, gonads, and pituitary [189]. 

#### 4.5.2. Clinical Characteristics

Initial symptoms of hemochromatosis like asthenia, joint pain, arthritis, chondrocalcinosis, diabetes mellitus, hypopituitarism, hypogonadotropic hypogonadism, cardiopathy, heart failure, and cardiac arrhythmias are uncharacteristic [221]. This is why patients are often seen by non-hepatologists including general practitioners, orthopedists, diabetologists, cardiologists, endocrinologists, and dermatologists for skin pigmentation, rarely and after long disease only. In addition, koilonychia, a nail disease that causes thin, flat, or concave nails, affecting the thumb and index finger has been observed in 50% of patients while 25% of patients tend to have it in all nails [189]. This is more common in the male gender, with an odds ratio of 4.3 (95% CI 2.97–6.18) for liver disease [27], as pre-menopausal females lose iron through blood physiologically via menstruation. This results in delayed disease detection in females, often reported in their sixth decade as compared with males in their fifth decade [189]. 

In hemochromatosis as seen by light microscopy, liver injury may start with steatosis [228] or mild steatohepatitis [229,230]. If not treated, the injury proceeds to chronic liver disease, fibrosis, cirrhosis, and eventually hepatocellular carcinoma (HCC) [229,231]. These initial stages are clinically not easily detectable by conventional laboratory parameters. Similarly, even when cirrhosis develops it is often a silent cirrhosis lacking symptoms, before clinical features of decompensated cirrhosis develop with jaundice, ascites, bleeding esophageal varices, and hepatic encephalopathy [228]. End stages of the natural course are acute liver failure, hepatocellular carcinoma, and death [27,228].

Using electron microscopy for assessing the liver of patients with hemochromatosis, injurious changes to the mitochondria and endoplasmic reticulum were described in necrotic hepatocytes with the highest liver iron content, associated with large iron-laden lysosomes in the hepatocytes that were also encountered in the Kupffer cells [232] in the pre-cirrhotic stage of idiopathic hemochromatosis; the first evident ultrastructural changes are in the lysosomal compartment. These changes correlate well with the iron overload also in advanced stages of the disease, and are reversed after iron removal.

#### 4.5.3. Diagnostic Approach

Initially, serum LTs like ALT and AST are normal or marginally increased up to two times the ULN [189,233], whereas later stages such as cirrhosis are clinically easier to recognize and show higher values of ALT, AST, and ALP [233]. Considering that mild abnormalities in the biochemical liver profile are common in hemochromatosis, patients with an unexplained abnormality in the liver profile should be screened for hemochromatosis with a serum ferritin and transferrin saturation [233]. Empirically, high values of serum ferritin and elevated transferrin saturation as well as genetic screening may help identify suspected hemochromatosis [221]. In general, a liver biopsy for histology examination is not required [231] and is no longer recommended [133]. Instead, the use of magnetic resonance imaging (MRI) allows for iron detection and quantification not only in the liver but also in other organs and has emerged as the reference standard imaging modality, as ultrasound is unable to detect iron overload and computed tomography findings are nonspecific and influenced by multiple confounding variables [231]. 

Despite many reports on the disease, there is no quantitative diagnostic algorithm available, which could provide information on the probability grades as to whether the diagnosis of hemochromatosis is likely, possible, or excluded [221]. Various diagnostic approaches were published, but none of the algorithms were based on evidence or quantitative results derived from scored key features [133,221,234]. 

#### 4.5.4. Therapy

RCTs in patients with hemochromatosis are lacking because venesection syn phlebotomy was introduced early as a first-line of therapy based on theoretical considerations that this therapeutic approach is the best way to efficiently remove an excess of iron from the body [133,235,236,237,238], and further research is considered as unlikely to change the confidence in the estimate of the benefit and risk of venesection in this disease [133]. Consensus exists that venesection is a first-line therapy for patients with hemochromatosis with similar proposals on how best to proceed [133,239]. As an example of qualification for venesection, patients should have a serum ferritin level >300 μg/L in men and >200 μg/L in women in combination with an elevated fasting transferrin saturation (>45%) for both men and women. Weekly or fortnightly venesection aims to achieve an initial serum ferritin target of 50–100 μg/L [238]. Each venesection typically would remove 500 mL of blood corresponding to 250 mg iron. Venesection intervals were increased, or a lower volume of blood was removed if the patient did not tolerate phlebotomy because of weakness, hypotension, or the development of anemia [238]. Venesection ameliorates overall prognosis but must be conducted as a lifelong therapy [133,235,236,237]. 

In a recent randomized crossover trial of erythrocytapheresis, which removes only red blood cells, versus phlebotomy, which removes blood with all cells, the conclusion was reached that erythrocytapheresis reduced the number of annual treatment procedures but at higher costs compared with venesection in the maintenance treatment of patients with HFE hemochromatosis [239]; erythrocytapheresis was subsequently classified as personalized erythrocytapheresis, representing the preferred treatment in selected cases [133].

As a second line of therapy in hemochromatosis for patients experiencing problems with venesection, iron chelation therapy using the once-daily, oral iron chelator deferasirox (Exjade) is available in selected patients [240,241] with contraindications for venesection such as anemia, severe heart disease, or poor venous access [240]. 

#### 4.5.5. Prognosis

Based on a large cohort consisting of 163 patients with hemochromatosis, cumulative survival was 92% at 5 years after diagnosis, 76% at 10 years, 59% at 15 years, and 49% at 20 years [235,236,237]. More specifically, life expectancy was lower in patients with cirrhosis as compared with those without cirrhosis, in patients with diabetes mellitus as compared with those without diabetes, and in patients who could not be depleted of iron during the first 18 months of venesection therapy as compared with those who could be depleted, while prognosis was not influenced by sex. On the other hand, patients without cirrhosis had a life expectancy that was similar to that expected in an age- and sex-matched normal population. Compared with the normal population, causes of death analyzed in fifty-three patients showed that hepatocellular carcinoma was two hundred nineteen times more frequent among the patients (sixteen patients), cardiomyopathy was three hundred six times more frequent (three patients), cirrhosis was thirteen times more frequent (ten patients), and diabetes mellitus was seven times more frequent (three patients). Finally, death rates for other causes, including extrahepatic carcinomas (seven patients), were similar to the rates expected. The conclusion was reached that patients with hemochromatosis diagnosed in the pre-cirrhotic stage and treated by venesection have a normal life expectancy, whereas cirrhotic patients have a shortened life expectancy and a considerable risk of liver cancer even when complete iron depletion has been achieved [235,236,237].

#### 4.5.6. Cascade of Molecular Events Leading to Iron Liver Injury

The pathogenesis of the iron liver injury was analyzed and discussed in previous reports [25,26,189,242,243]. The focus is on hemochromatosis, including intoxications by overdosed iron, in patients but rarely in animal studies. 

The first pathogenetic step in hemochromatosis causing liver injury can be traced back to genetically triggered overwhelming iron absorption through the intestinal tract [169] specifically in the human upper small intestine microvillous membrane vesicles [170] and in the rat model [171,172]. This causes a toxic iron overload of the human organism including the liver [133,236,237,238,243,244,245,246]. Hemochromatosis is an autosomal recessive disorder caused by mutations in genes involved in iron metabolism, which results in increased intestinal iron absorption [246]. The abnormally high intestinal uptake of iron is likely due to mutations in several genes, including HFE, transferrin receptor 2 (TFR2), hepcidin, ferroportin (SLC40A1), and hemojuvelin (HFE2) [189,220,242,245], with a preference for the HFE gene that encodes the HFE protein [220,244]. This protein normally regulates the production of hepcidin responsible for iron homeostasis and especially, the control of iron uptake by cells through its interaction with the transferrin receptors, processes that do not function in patients with hemochromatosis [220]. This is shown by the downregulation of hepcidin synthesis, leading to increased intestinal iron absorption [244]. Thus, mutations in the HFE gene lead to excess iron absorption and iron overload in hemochromatosis [232]. 

The second mechanistic step represents the excessive iron uptake by the hepatocytes from blood, where it is transported by transferrin [228,246,247]. This follows the intestinal iron uptake as the first step of the cascade of events leading to liver injury [248].

The third step in hemochromatosis occurs in the liver itself with iron overloaded hepatocytes. It starts with injurious attacks of iron as divalent ferrous iron (Fe^2+^), a cation capable of reacting with hydrogen peroxide, generating one of the reactive oxygen species (ROS), the hydroxyl radical, while being oxidized to Fe^3+^ [228]. Radicals generated in the so-called Fenton–Haber–Weiss reaction are known as some of the most dominant oxidants found in the human body, attacking proteins, lipids, nucleic acids, and carbohydrates and leading to peroxidation and cell apoptosis [167,248,249,250]. ROS injure membranes of subcellular organelles, such as mitochondria or the endoplasmic reticulum, which contain structural proteins and phospholipids with polyunsaturated fatty acids (PUFAs) that are peroxidized, as evidenced by lipid peroxide markers found in patients with hemochromatosis or overloaded by exogenous iron [249,251,252]. As an example, in patients with hemochromatosis, liver biopsy specimens were immunostained for protein adducts with malondialdehyde and 4-hydroxynonenal, and both adducts were found to be more abundant as compared with controls, whereby the staining had a predominance in acinar zone 1 that followed the localization of iron [253]. Similarly, enhanced oxidative stress was described in patients with hemochromatosis, as evidenced by hepatic malondialdehyde (MDA)-protein adducts and by increased oxidatively modified serum proteins [251]. MDA-lysine epitopes and oxidatively modified serum proteins, as well as immunoglobulin G autoantibodies against MDA-lysine epitopes, were increased in untreated hemochromatosis patients compared with normal individuals, and these markers of ongoing oxidative stress decreased with phlebotomy treatment in hemochromatosis patients. In addition, TGF-beta1 colocalized with hepatic iron and MDA protein adducts in hepatocytes and sinusoidal cells of hepatic acinar zone 1 and normalized after iron removal. Iron overload increases both lipid peroxidation and TGF-beta1 expression, which together could promote hepatic injury and fibrogenesis leading to liver fibrosis and finally cirrhosis [251].

Ferroptosis, an iron-dependent form of regulated cell death [254,255,256,257], is closely related to mechanistic sequalae described in conditions of iron overload like hemochromatosis [176,256,257], and was discussed in detail more recently [257]. There is some evidence that ferroptosis is triggered by ferritinophagy, an autophagic process that specifically involves ferritin to release intracellular free iron [257]. At the morphological level, ferroptosis causes injury to mitochondria, with condensed, ruptured outer membranes, associated with initial iron accumulation, excessive ROS production, and excessive lipid peroxides [26,257]. Polyunsaturated fatty acids (PUFAs), as structural components of membrane phospholipids, are easily peroxidized, forming lipid peroxides triggered by ROS, generated through the Haber–Weiss reaction. Ferroptosis is also involved in alcoholic liver injury via liable iron accumulation and in the associated hepatic glutathione exhaustion [257]. 

The fourth step addresses the possible role of cytokines in hemochromatosis. The liver is commonly a secret-keeping organ providing few markers like cytokines or chemokines in the blood for a quick analysis of mechanistic processes within the liver in the context of liver injury. In patients with hemochromatosis, several cytokines were analyzed including IL1α, IL1β, IL2, IL4, IL6, IL8, IL10, IL12, IL17, IFNγ, TNFα, and Gm-CSF, which could help investigate the inflammatory status of the study population. As opposed to none of the individuals in the control group, serum IL8 was elevated in 42% of C282Y homozygotes and 46% of H63D patients, as homozygotes or in combination with C282Y [245]. The observation that several hemochromatosis patients had elevated levels of IL8 is difficult to explain but may be due to the fact that the C282Y HFE protein induces the transcription factor NF-κB, which consequently resulted in a marked increase in the protein production of IL8 along with an increased transcriptional activation of IL8 in C282Y HFE-expressing cells [245,258]. Elevated levels of IL8 were especially observed in patients who had recently been treated with phlebotomy, which might suggest a relation between disease severity and levels of IL8 [245]. Similar to other cytokines, IL8 as a chemokine is produced by macrophages and other cell types such as epithelial cells and airway smooth muscle cells [259], and can be significantly upregulated in patients harboring the H63D mutation; conceptually, however, the causes of neither the increased serum IL8 levels nor the unchanged levels of the other cytokines observed in hemochromatosis patients were thoroughly investigated, and these results currently do not add to our understanding regarding mechanistic steps involving mediators that are secreted by non-parenchymal cells within the liver and may interact among each other [167,245]. The failure to detect many cytokines [245] may be ascribed to the fact that the respective studies were carried out in patients with advanced stages of hemochromatosis already requiring phlebotomy therapy, rather than in those with the early phases of the disease. As an alternative, the immune cells commonly secreting cytokines under normal conditions may have so heavily been injured by the iron that they no longer produced and secreted cytokines. 

Finally, the fifth mechanistic step has a focus on the gut microbiome, which is altered in hemochromatosis patients [238]. In this study cohort, systemic iron reduction by phlebotomy was associated with an alteration of the gut microbiome, with changes evident in those who experienced reduced fecal iron availability with venesection. For example, levels of *Faecalibacterium prausnitzii*, a bacterium associated with improved colonic health, were increased in response to fecal iron reduction. During iron depletion, iron absorption from the gastrointestinal tract increases to compensate for iron lost with treatment. Consequently, iron availability is limited in the gastrointestinal tract and is crucial to the growth and function of many gut bacteria. Moreover, increased colonic iron has been associated with colonic inflammation and oxidative stress. Similarly, metabolomic changes were seen in association with reduced fecal iron levels, with significant changes in microbial metabolites after treatment, when increases in pyruvate, tyrosine, methionine, glycine, and aspartate were observed. In these patients, there was a greater separation in the metabolome, where a shift was observed towards a more positive metabolomic profile with treatment compared with baseline [238].

**Figure 5:**
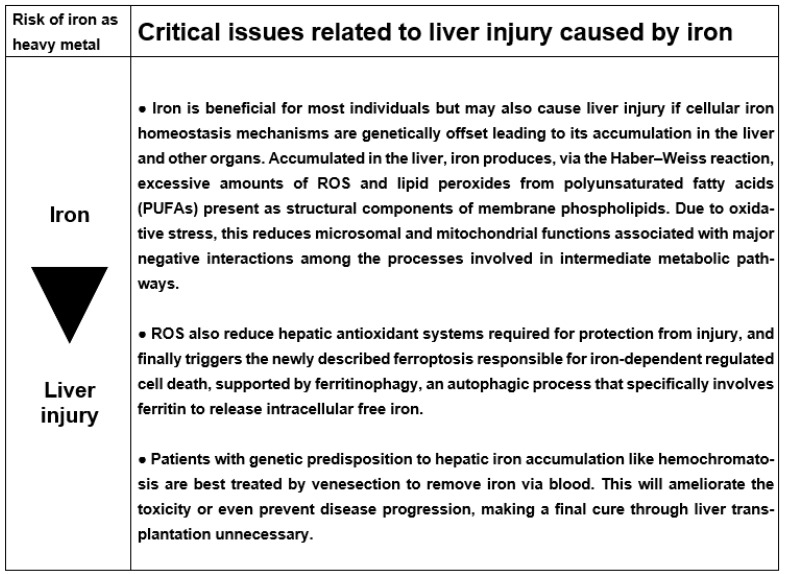
Listing of key issues related to liver injury caused by iron. Abbreviation: ROS, reactive oxygen species.

The main critical issues related to liver injury caused by iron were discussed and quoted before [254,255,256,257] with a special focus on ferroptosis [257], and are summarized (Figure 5). 

## 5. Cadmium

Cadmium (Cd) (from ancient Greek καδμία kadmía or Latin cadmia) is environmentally present [2,260,261,262,263], and is encountered by humans [263].

### 5.1. Physiology 

Cadmium in its ionic form of Cd2+ is a non-essential divalent metal and toxic in eukaryotic cells [264]. Consequently, cadmium is not required for any biological process involving, for instance, enzymes. Hence, there is no physiological downsizing of this metal once it enters the human body because mechanisms of ensuring cadmium homeostasis and that would control its uptake and release are missing. Actually, infants may already have cadmium in their body, provided by their mothers, since cadmium crosses the placental barrier and easily reaches the fetus [265]. In this context, cadmium was found in the cord blood, maternal blood, and placental tissue [266]. With advancing age and a continuous intake of cadmium via the lungs and the gastrointestinal tract [267], there is an increasing accumulation of cadmium in the body, where it may remain for 20 to 30 years as a non-biodegradable metal [263,267]. 

The presence of substantial amounts of cadmium accumulated in kidney, liver, bones, eyes, and other organs suggests an effective intestinal absorption of cadmium derived from ingested food contaminated with environmental cadmium and a functioning systemic transport of cadmium [268]. This is accomplished by employing multiple cellular transporters that are primarily used for the acquisition of the calcium, zinc, and manganese needed by the body. 

### 5.2. Cadmium as Pollutant and the Issue of Health Hazards

#### 5.2.1. Sources of Cadmium as an Environmental Pollutant

Cadmium is regularly found together with other heavy metals such as zinc, copper, and lead [269], detectable ubiquitously in the environment [23,25,26,30,270,271,272]. Overall environmental cadmium pollution is well documented using the excellent attic dust approach, whereby dust collected from the attic of old houses is examined, providing an archive of historical air contamination by cadmium in the urban environment [71]. 

Environmental cadmium concentrations continuously increase due to soil disruption, earth volcanic activity, and as byproduct of industrial processes [30,269,272] because it is used in alloys, pigments, stabilizers, and batteries [30]. Industrially, its use is best known for electroplating and in the production of nickel-cadmium batteries [269]. Consequently, cadmium is a contaminant of municipal solid waste landfills [23,270], electronic waste [273], soils, and plant parts such as leaves, stems, and roots [23]. Human exposure may occur via inhalation, including cigarette smoking, as well as by food [30], such as plants and vegetables contaminated [23] through the uptake of cadmium via roots or rhizome or via horizontal transfer (Figure 3), including potatoes, grains, seeds, or mushrooms, in addition to crustaceans, mollusks, shellfish, mussels, cocoa powder, and dried seaweed [30]. 

#### 5.2.2. Elucidating Health Hazards of Environmental Cadmium

The widespread occurrence of the pollutant cadmium and the inability of humans exposed to this heavy metal to safely remove it from their body explains the broad interest of scientists and physicians in the health hazards possibly caused by cadmium (Table 5) [263,268,269,274,275,276,277,278,279,280,281]. 

Food, water, and air contaminated with environmental cadmium represents the major health hazard for exposed humans (Table 5) [263,268,269,274,275,276,277,278,279,280,281]. As cadmium is not biodegradable in the human body, it accumulates and exerts its toxic effects on a variety of organs, which leads to functional impairment including the liver with increased LTs in one study [280] but not in two others [269,279]. Under clinical aspects and much more seriously, however, prolonged exposure to cadmium is associated with increased mortality due to cancer and cardiovascular diseases [281]. Despite worldwide efforts to reduce cadmium emission in the environment, cadmium exposure to humans is largely unavoidable as measures to reduce cadmium intake are not really effective. 

In essence, when finally entering the earth after being generated in the universe, cadmium is not an essential element in humans, who are not prepared to provide conditions for maintaining cellular cadmium homeostasis. Instead, cadmium is a typical pollutant responsible for many diseases due to unwanted continuous uptake and storage in the human body. 

### 5.3. Acute Human Liver Injury by Cadmium

Acute cadmium intoxications in humans commonly occur by inhalation, resulting in lung injury, while liver injury has rarely been reported following the ingestion of cadmium salts [206,282,283,284], leaving liver values unchanged [284] or causing slightly increased LTs [283]. In one patient, the leading symptom was an initially unexplained hemolytic anemia with a complicated clinical course finally leading to death due to multiorgan failure. ALT activity was 86 U/L (normal range 5–45 U/L) thereby excluding a severe grade of liver injury, confirmed by an autopsy that showed by histology only diffuse centrilobular congestion and necrosis of the liver and by cadmium quantification low levels similar to the heart and brain, whereas extremely high cadmium levels were found in the kidneys, explaining the renal failure that required dialysis [283]. For the ingestion of cadmium, activated charcoal and gastric lavage are options in addition to mandatory monitoring for gastrointestinal injury as well as renal and hepatic dysfunction [269]. Chelators, though promising, have not been definitively proven to be useful in cadmium toxicity. However, it appears that if succimer is given early in the course, it has promising results. A comparison of the effectiveness of several chelators was published after a single experimental cadmium administration evaluating the toxicity, excretion, and distribution of cadmium [269,285]. 

### 5.4. Chronic Human Liver Injury by Cadmium

Serum LTs such as ALT, AST, and ALP in humans following prolonged cadmium exposure were commonly reported as having variable and mostly low values, often interpreted by the authors as signs of liver injury or liver disease [279,286,287,288,289,290,291,292]. However, published LT values did not commonly meet diagnostic criteria and thresholds of real liver injury as required [33], and occasionally normal ranges of the listed parameters were not provided. Low LT values in these patients are either due to low cadmium exposure or may reflect liver diseases that have nothing to do with cadmium, as causality assessment using RUCAM was not performed. Based on association studies analyzing serum cadmium levels, there is also the proposal that cadmium may cause hepatic steatosis and fibrosis [288], classified as non-alcoholic fatty liver disease (NAFLD) and non-alcoholic steatohepatitis (NASH) [289], now known as metabolic dysfunction-associated fatty liver disease (MAFLD) and metabolic dysfunction-associated steatohepatitis [292,293]. 

Chronic exposure to cadmium was described as related to positive associations with liver damage. Light microscopy of the liver specimens obtained from patients with chronic cadmium exposure showed significantly more fibrosis and hemosiderosis as compared with nonexposed controls [293]. Fibrosis was periportal, portal, perisinusoidal, or bridging. Additional features included inflammation, hepatitis, and macro- or microvesicular steatosis. These light microscopy changes in humans were grossly confirmed in corresponding animal models [294,295]. In other experimental microscopy studies, diffuse hepatocellular degeneration and necrosis were observed, also described as hemorrhagic lesions and coagulative necrosis, associated with high plasma ALT activity and high cadmium concentration in the liver [296]. The reported vacuolar degeneration was not further discussed but may represent vacuoles corresponding to empty fat cells following staining procedures with hematoxylin and eosin. In addition, other histopathological observations confirmed extensive parenchymal degeneration [297]. 

Transmission electron microscopy showed the presence of autophagosomes and dilated endoplasmic reticulum, which represents the microsomal fraction of the biochemists, and translucent vesicles and structural changes of the liver comprised the sinusoids being replaced by large gaps [298]. Additional ultrastructural studies focused on the proliferation of the endoplasmic reticulum and lysosomes and confirmed the presence of autophagosomes [296,298]. 

### 5.5. Cascade of Molecular Events Leading to Cadmium Liver Injury

The mechanism of liver injury caused by cadmium was discussed in several reports [25,26,264,279,299]. Most suggestions were derived from animal studies while respective data on patients were in the minority. 

The first mechanistic step leading to liver injury after cadmium uptake by the human body and arrival in the circulation starts with with cadmium entering the liver; this is a complex approach because hepatocytes can only be targeted by Cd^2+^ if it hijacks physiological high-affinity entry pathways [264]. They help transport essential divalent metal ions in a process termed “ionic and molecular mimicry”. Hence, “free” Cd^2+^ ions and Cd^2+^ complexed with small organic molecules are transported across cellular membranes via ion channels, carriers, and ATP-hydrolyzing pumps, whereas receptor-mediated endocytosis internalizes Cd^2+^–protein complexes [264]. Within the liver cell, the ionic form of Cd^2+^ binds to the protein metallothionein (MT) to form the cadmium metallothionein (Cd–MT) complex, which allows for cadmium deposition in the liver and downgrades glutathione production [271].

The second step involves the covalent binding of Cd^2+^ to sulfhydryl groups on critical molecules such as hepatic mitochondria and cytosolic glutathione [271,299,300]. 

The third step is characterized by the reduced antioxidant function of the cytosolic glutathione and the overwhelming mitochondrial oxidative stress causing lipid peroxidation, which can no longer be mitigated by glutathione, contributing to the overall cellular production of ROS in hepatocytes detected by mass cytometry and fluorescence microscopy concomitantly with cadmium uptake [300]. An experimental exposure of hepatocytes to cadmium increased the concentration of malondialdehyde, a marker of lipid peroxidation, and decreased the activity of antioxidant enzymes, such as superoxide dismutase, catalase, glutathione reductase, and glutathione peroxidase [301]. Ferroptosis is involved in liver injury by cadmium, accompanied by the activation of the PERK-eIF2α-ATF4-CHOP signaling pathway, and ferroptosis in cadmium liver injury is dependent on endoplasmic reticulum stress [295]. The gut is also a target for experimental cadmium toxicity modulating the gut microbiome but studies in humans with the respective liver injury are not available, likely because of the small number of cases [302]. 

Fourth, experimental liver oxidative stress induced by cadmium was also ascribed to the activation of hepatic stellate cells [299]. 

The fifth step causing the injury may be triggered by cadmium causing the infiltration of polymorphonuclear neutrophils and Kupffer cells into the liver [303]. Cytokines and chemokines produced by activated Kupffer cells, such as tumor necrosis factor-alpha, interleukin-1, and interleukin-6, have been shown to be associated with inflammation and subsequent liver injury [299,300,304].

The last step of the liver injury focuses on the role of cadmium in modulating autophagy and tumorigenesis including hepatocellular carcinoma [271]. A recent meta-analysis proposed elevated serum and hair levels of cadmium as a risk factor for hepatocellular carcinoma [305].

**Figure 6:**
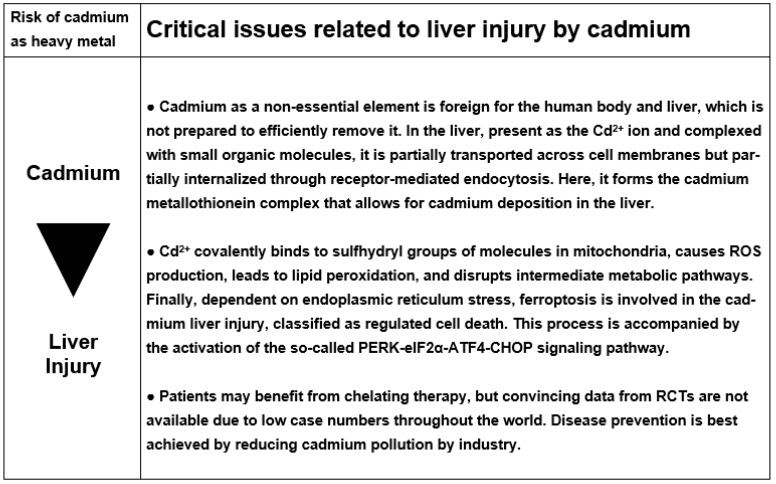
Relevant challenges related to liver injury due to cadmium. Abbreviations: RCTs, randomized controlled trials; ROS, reactive oxygen species.

The key critical issues related to liver injury caused by cadmium are summarized (Figure 6).

## 6. Arsenic

Arsenic (As, from Latin arsenicum) formed in the universe is environmentally present and potentially hazardous for human health [2,260,261]. 

### 6.1. Physiology

Arsenic is a non-essential element for human health because it is without a known nutritional or metabolic role and is not a constituent of any enzyme [306,307]. Similar to other non-essential metals like cadmium, arsenic, lead, nickel, barium, chromium, and mercury, it is harmful to health even at low concentrations [25,26,306]. Arsenic is commonly consumed together with food and affects intestinal homeostasis by disrupting barrier function and inducing inflammatory responses [308]. Based on experimental studies, processes involved in the transit of arsenic through the gastrointestinal tract can affect toxicity, as the more toxic of the ingested arsenic species may be formed in the intestinal lumen resulting from an interaction of arsenic with food components or gut microbiota, conditions that may modulate arsenic intestinal absorption and its adverse effects [308]. The experimental intestinal absorption of arsenic is variable and depends on the arsenic species used and the function of a possible paracellular transport [308]. Having no known cellular transporter system, arsenic is taken up into the cell by the phosphate carrier because arsenic is a phosphate analogue [307]. This process likely occurs in humans intoxicated with arsenic, which has been found in the liver and kidneys [309,310,311,312,313] with a maximum arsenic content of 6 mg/kg in the liver [310]. In this context, the normal human body contains 0.02–0.08 mg arsenic/kg body weight, which is concentrated in the liver, kidneys, lungs, bones, and hair [309].

### 6.2. Arsenic as Pollutant and the Issue of Health Hazards

Arsenic originating from the universe is frequently found in the environment of our earth [26,309,311,312,313] including the atmosphere originating from natural sources such as volcanic eruptions and industrial processes [313]. This metalloid is also a contaminant of soils [309,314], surface water [309,315], and ground water used as drinking water [310,313,316], and food [313,316]. In addition, arsenic was used in chemical warfare agents [309,313] and is a known component of some conventional or traditional medicines [24,309,311,313,317,318,319,320,321,322] including those used in Ayurveda medicine [24,320,323,324,325,326,327] and herbal Traditional Chinese Medicine (TCM) [24,328,329,330,331]. However, arsenic is not necessarily a common pollutant in municipal solid waste landfills [24]. Finally, environmental arsenic pollution is a longstanding phenomenon as evidenced by the attic dust approach, whereby dust collected from the attic of old houses is examined, providing an archive of historical air contamination by arsenic in the urban environment [71]. 

#### 6.2.1. Sources of Arsenic as an Environmental Pollutant

Arsenic exists as a metalloid (As^0^) [311] and is found in the environment as the inorganic As(III), arsenite [311,332], which is more toxic than As(V), arsenate [332]. Other forms include organic arsenic and arsine (AsH_3_) [311]. Reviewing the abundant reports, arsenic shows variable degrees of toxicity with As_2_O_3_ as one of the most powerful poisons [306,307,308,309,310,311,312,313,314,315,316,317,318,319,320,321,322,323,324,325,326,327,328,329,330,331,332]. The liver is the primary target organ not only for toxicity but also for the metabolism of arsenicals with its major metabolic pathway being via methylation [311,332], which leads to the methylated intermediates of monomethylarsonic acid (MMA) and dimethylarsinic acid (DMA) [332]. 

#### 6.2.2. Elucidating Health Hazards of Environmental Arsenic

Potential health risks have been reported in various publications, a selection of these are listed (Table 6) [310,333,334,335,336,337,338,339,340,341,342,343,344,345,346,347,348,349,350,351,352,353,354,355,356].

Increased LTs due to a chronic intake of arsenic together with tap water or food have been published but the causal attribution of arsenic to real liver injury often remained unclear [310,332,357,358,359]. In general, increases were marginal with serum ALT values of 36.60 ± 11.07 U/L in exposed individuals vs. 31.80 ± 9.41 U/L in nonexposed ones [357] and much lower than those required for a significant liver injury [33]. Similarly, individuals were included with ALT values >1 times the ULN, again not representative for a significant liver injury [310]. These cohorts with marginally increased LTs [310,357] and partially associated clinical signs of hepatomegaly [310] should instead be interpreted as harmless liver adaptation syn liver tolerance because LTs commonly experience a spontaneous return to normal values even if the intake of the causative chemical is not stopped. There is also concern because efforts to exclude alternative causes unrelated to arsenic intake are limited. As individuals are exposed not only to arsenic alone but often also concomitantly to other heavy metals and toxins, causality attribution to arsenic may become a matter of debate. In an early study comprising 2248 patients with evidence of chronic arsenic toxicity, hepatomegaly was present in 190 of 248 patients (76.6%), non-cirrhotic portal fibrosis was the predominant lesion (91.3%) in liver histology, and the maximum arsenic content in the liver was 6 mg/kg, but it was undetected in six of twenty-nine samples studied [310]. Despite diagnostic shortcomings, sufficient evidence exists that a prolonged intake of environmental arsenic may cause liver injury (Table 6) [310,332,357,358,359]. 

Apart from targeting the liver, arsenic can jeopardize multiple organs (Table 6), a process that develops through various pathways, influenced by environmental bioprocesses [352]. In general, chronic exposure to arsenic may cause malignant, degenerative, and inflammatory diseases [339,345]. As a consequence, these arsenic-related disorders increased the disability-adjusted life years (DALYs) [350] and the mortality rates secondary to specific cancers [336]. 

### 6.3. Acute Human Liver Injury by Arsenic

Acute intoxication by a suicidal ingestion of around 8 g arsenic trioxide, which the patient found in the pharmacy of his grandmother, resulted in a fatal outcome and high arsenic amounts in the liver with 147 µg/g dry weight and As^3+^ as the predominant form, with a higher concentration of MMA compared with DMA, followed by the kidneys with 26.6 µg/g [360]. In other cases of acute arsenic intoxication, ante-mortem blood and urine concentrations ranged from 2.3 to 6.7 µg/mL [361]. 

Autopsy examinations in patients with acute arsenic poisoning revealed fat degeneration of the liver [362], or more specifically, microvesicular steatosis [363]. After a massive overdose of arsenic trioxide, LT were only marginally elevated with a serum AST activity of 51 U/L and ALT of 68 U/L [364]. 

Clinical symptoms of acute arsenic poisoning classically start with gastroenteritis involving diarrhea like “rice water” that may be bloody and is associated with severe hypotension as another hallmark viewed as secondary to dehydration and volume loss [312]. Other features may include colicky abdominal pain, arrhythmias, proteinuria, hematuria, and acute renal failure, as well as headache, delirium, encephalopathy, seizures, and a metallic taste occuring in the mouth with a slight odor of garlic in the breath [309]. Typical clinical signs of liver injury like jaundice or increased serum conjugated bilirubin levels are rarely observed [312] or not reported [309]. Acute arsenic exposure is confirmed at a level of 50 µg/L in spot urine or over 100 µg of total arsenic in a 24 h collection using a metal-free polyethylene container [312]. In acute exposure, 24 h urinary arsenic levels typically exceed several thousand micrograms. Spot urine levels commonly exceed 1000 µg/L. An abdominal radiograph may demonstrate intestinal radiopaque metallic flecks in arsenic ingestion [312].

Therapy using metal chelators must be quickly initiated at the first suspicion of an arsenic intoxication without waiting for urinary arsenic results [309,312]. Under consideration and thoroughly discussed are thiol chelators such as meso-2,3-dimercaptosuccinic acid, 2,3-dimercaptopropanol syn sodium 2,3-dimercapto-1-propanesulfonate known as the British-Lewisite antidote (BAL), penicillamine, and ethylenediaminetetraacetic acid, which provide the formation of inert chelator–metal complexes and help through the urinary excretion of arsenic from the body. Other measures include gastrointestinal lavage via mouth or large bowel lavage via rectum, based on anecdotal reports lacking evidence from RCTs due to small case numbers. This limitation also applies to any gastric bezoar suspected to consist of arsenic material upon radiology assessment that needs removal from the stomach by laparotomy or, currently preferred, by endoscopy. Lethality follows in the first few hours from shock or days later from acute renal or liver failure [365].

### 6.4. Chronic Human Liver Injury by Arsenic

Prolonged arsenic uptake by humans causes the toxicity of various organs including the liver [309,313,316,366] and leads to clinical features due to arsenic poisoning [38,309,313,317,318]. Abnormal LTs like serum activities of ALT, AST, and ALP have been published [272,318,319,321,332] with significantly higher values in patients exposed to substantial amounts of arsenic [332]. As an example, increased LTs were documented in two patients on a therapy including herbal, arsenic-containing Ayurvedic medicines: the first patient (68 years old, male) was on the arsenic-containing remedy for 32 days and showed increased LT values of ALT 473 U/L, AST 498 U/L, and ALP 188 U/L, whereas the second patient (48 years old, female) was on the arsenic medication for 10 days and showed LT increases of ALT 1122 U/L, AST 898 U/L, and ALP 364 U/L. Both cases were assessed for causality using the updated RUCAM [319] and were discussed regarding data interpretation [325,367]. 

Light microscopical liver histology in patients with prolonged arsenic uptake is characterized by liver fibrosis leading to clinical non-cirrhotic portal hypertension [368,369,370,371], whereby studies on the wedged hepatic vein pressure indicated that the obstruction to portal flow resided in the portal tracts [369]. Histology features of the liver include portal tract fibrosis [368,369,371] and an increase in the number of portal veins with thickening and hypertrophic walls in some reports [368,369]. A few inflammatory cells were described and mild fatty change was seen in the surrounding hepatocytes [368], a finding subsequently confirmed by electron microscopy [317]. 

### 6.5. Cascade of Molecular Events Leading to Arsenic Liver Injury

Pathogenetic sequalae involved in liver injury caused by arsenic were preferentially derived from animal studies and case reports, analyzed and reviewed in recent articles [25,26,309,311,312,372]. According to mainstream opinion, several steps are under discussion. 

The first step depends on the active transport of the protein-bound arsenic from the blood into the liver. This is facilitated by anion exchange transporters in the plasma cell membrane of hepatocytes, in which arsenic replaces the phosphate [307]. As a non-essential element in humans, arsenic has no specific transporter system on its own. 

The second step of the cascade of events is characterized by the binding of arsenic in its most toxic form of As^3+^ with sulfhydryl groups of enzymes and membrane proteins within the hepatocytes, conditions that lead to cross linkage [311,332,373,374]. Contributing factors to arsenic liver injury are oxidative DNA, acquired tolerance to apoptosis, enhanced cell proliferation, altered DNA methylation, and genomic instability [332,375]. Most of these alterations were confirmed in an experimental model using zebrafish (*Danio rerio*) intoxicated with arsenic and evaluated for metabolomic changes [376]. 

The third and final step considers, among others, the replacement of phosphate with arsenic in enzymes involved in the mitochondrial anion exchange transport system Na^+^/K^+^-ATPase, which impairs the function of the respiratory chain, as well as the binding of arsenic to the sulfhydryl groups of the ATPases [6,377,378]. 

Arsenic caused experimental liver injury via ROS, whereby specific radicals are generated [379,380,381], partially by mechanisms including CYP (Figure 3) [379]. Among the radicals were the nitric oxide NO•, singlet oxygen 1O2, hydrogen peroxide, and dimethylarsinic peroxyl radical (CH3)2AsOO•. The individual molecular steps leading to these radicals are difficult to firmly establish because the processes are fast and do not allow capture. However, ROS formation was experimentally ascertained because glutathione attenuated arsenic liver injury as evidenced by ameliorating the ROS parameters like malondialdehyde, superoxide dismutase, 8-hydroxy-2′-deoxyguanosin, and glutathione peroxidase [382]. 

In experimental studies, arsenic causes an imbalanced immune response as verified by the notable abnormal ratio of Th17 to Treg cells in peripheral blood as well as the secretion of inflammatory cytokines IL-17A, IL-6, TGF-β1, and IL-10 in the serum and liver [383]. In humans, a systematic review and meta-analysis revealed that inflammatory cytokines like interleukin (IL)-6, IL-8, and IL-12 levels were significantly higher in arsenic-exposed individuals compared with the control group, indicating that arsenic exposure can cause inflammatory responses [384].

**Figure 7:**
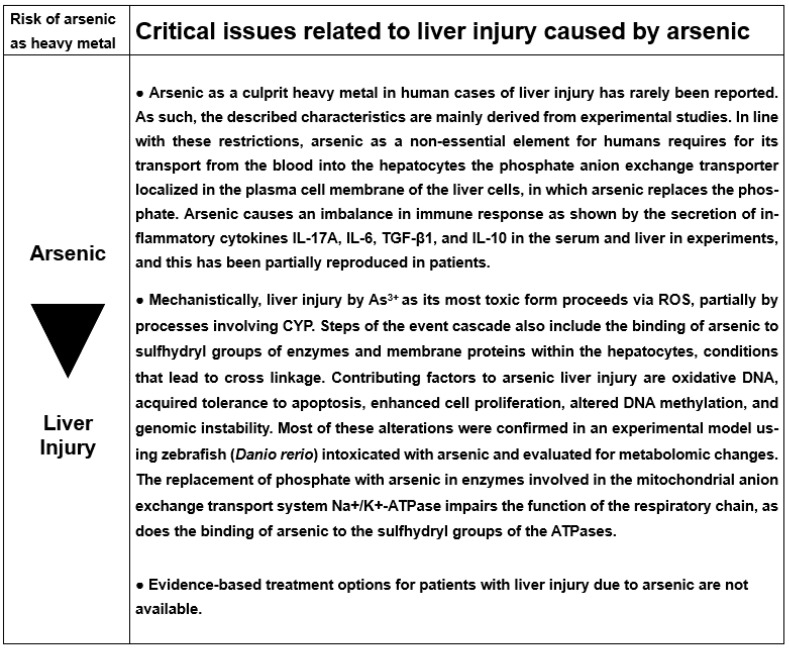
Selected critical issues of liver injury due to arsenic. Abbreviations: CYP, cytochrome; ROS, reactive oxygen species.

The key critical issues related to liver injury caused by arsenic are presented in condensed form (Figure 7). 

## 7. Conclusions

Heavy metals share with other substances, like per- and polyfluoroalkyl substances (PFAS), the common characteristics of eternity, persistence, and issues of complete biodegradability that may be hazardous to human health. However, whereas both substance categories are derived from humankind and industry activities, heavy metals also have part of their origin in nature before portions thereof entered the industry segment. As shown in this analysis, heavy metals like copper, iron, cadmium, and arsenic may differently modify human health resulting in positive, negative, or mixed effects. More specifically, liver injury may become a key feature in individuals, if these heavy metals accumulate in the liver. This potentially dangerous accumulation is the result of limited mechanisms providing the required cellular heavy metal homeostasis through the control of uptake and excretion. For instance, genetic mutations cause copper accumulation in the liver of patients with Wilson disease or the same for iron in patients with hemochromatosis. As opposed to and unrelated to any genetic abnormality, cadmium and arsenic constantly accumulate during the whole lifespan in the liver, which is unable to provide the specific cellular homeostasis required. At the mechanistic level, any heavy metal may generate toxic radicals in excess amounts that are insufficiently detoxified through hepatic antioxidant capacities, which are exhausted in the meantime and thereby unable to prevent liver injury. Causality attribution for a single culprit heavy metal may be difficult as they are mostly encountered by humans not singly but in combination with other elements. In this context, the exclusion of alternative acute or chronic liver diseases is mandatory to clinically eliminate confounders. Humans occasionally tend to disqualify heavy metals globally, although essential heavy metals, with copper and iron as examples, among others, were used for human evolution and are still urgently needed for maintaining human health. Their benefits outweigh the risks by far, in addition to their role in ensuring the evolution of humankind, fauna, and flora. Without essential heavy metals, humankind would not exist. So, global criticism on these elements is not really warranted. Genetic disorders like Wilson disease or hemochromatosis can be treated well if recognized early, whereas health hazards due to cadmium or arsenic and other heavy metals are principally preventable diseases through avoiding or reducing their uptake but essentially require more regulatory efforts to help reduce their industrial emissions.

## Figures and Tables

**Table 1:** Differential diagnoses of toxic liver injury.

Differential Diagnosis	Diagnostic Parameters
Hepatitis A virus (HAV)	Anti-HAV IgM
Hepatitis B virus (HBV)	HBV DNA, anti-HBc IgM
Hepatitis C virus (HCV)	HCV RNA, anti-HCV
Hepatitis E virus (HEV)	HEV RNA, titer change for anti-HEV IgM/anti-HEV IgG
Cytomegalovirus (CMV)	CMV PCR, titer change for anti-CMV IgM/anti-CMV IgG
Epstein Barr virus (EBV)	EBV PCR, titer change for anti-EBV IgM/anti-EBV IgG
Herpes simplex virus (HSV)	HSV PCR, titer change for anti-HSV IgM/anti-HSV IgG
Varicella zoster virus (VZV)	VZV PCR, titer change for anti-VZV IgM/anti-VZV IgG
Other viral infections according to the clinical context	Specific serology of Adenovirus, Coxsackie-B-Virus, Echovirus, Measles virus, Rubella virus, Flavivirus, Arenavirus, Filovirus, Parvovirus, HIV, and others
Other infectious diseases	Specific assessment of bacteria, fungi, parasites, and others
Autoimmune hepatitis (AIH) type I	Gamma globulins, ANA, SMA, AAA, SLA/LP, anti-LSP, and anti-ASGPR. Simplified criteria for the diagnosis of autoimmune hepatitis.
Autoimmune hepatitis (AIH) type II	Gamma globulins, anti-LKM-1 (CYP 2D6), anti-LKM-2 (CYP 2C9), and anti-LKM-3. Simplified criteria for the diagnosis of autoimmune hepatitis.
Primary biliary cholangitis (PBC)	AMA, anti-PDH-E2
Primary sclerosing cholangitis (PSC)	p-ANCA, MRC
Autoimmune cholangitis (AIC)	ANA, SMA
Overlap syndromes	See AIH, PBC, PSC, and AIC
Non-alcoholic steatohepatitis (NASH)	BMI, insulin resistance, hepatomegaly, echogenicity of the liver
Alcoholic liver disease (ALD)	Patient’s history, clinical and laboratory assessment, other features of alcoholic disease
Cocaine, ecstasy and other amphetamines	Toxin screening
Rare intoxications	Toxin screening for household and occupational toxins
Drug-induced liver injury (DILI) and herb-induced liver injury (HILI)	Diagnostic algorithm of the updated Roussel Uclaf Causality Assessment Method (RUCAM) for DILI and HILI
Hemochromatosis	Serum ferritin, total iron-binding capacity, genotyping for C2824 and H63D mutation, hepatic iron content
Wilson disease	Copper excretion (24-h urine), ceruloplasmin in serum, free copper in serum, Coombs-negative hemolytic anemia, hepatic copper content, Kayser–Fleischer ring, neurologic-psychiatric work-up, and genotyping. Modified Leipzig Scoring System for diagnosis of Wilson disease.
Porphyria	Porphobilinogen in urine, total porphyrins in urine
α1-Antitrypsin deficiency	α1-Antitrypsin in serum
Biliary diseases	Clinical and laboratory assessment, hepatobiliary sonography, and other imaging (CT, MRC)
Pancreatic diseases	Clinical and laboratory assessment, sonography, CT, MRT
Celiac disease	TTG antibodies, endomysium antibodies, duodenal biopsy
Anorexia nervosa	Clinical context
Parenteral nutrition	Clinical context
Cardiopulmonary diseases	Cardiopulmonary assessment of congestive heart disease, myocardial infarction, cardiomyopathy, cardiac valvular dysfunction, pulmonary embolism, pericardial diseases, arrhythmia, hemorrhagic shock, and various other conditions
Addison disease	Plasma cortisol
Thyroid diseases	TSH basal, T4, T3
Grand mal seizures	Clinical context of epileptic seizure (duration >30 min)
Heat stroke	Shock, hyperthermia
Polytrauma	Shock, liver injury
Systemic diseases	Specific assessment of sarcoidosis, amyloidosis, metastatic tumor, sepsis, and others
Other diseases	Clinical context

This flow chart compilation is adapted and derived from a publication, and although not comprehensive, it is to be used as a guide for patients with suspected liver injury [33]. Abbreviations: AAA, anti-actin antibodies; AMA, antimitochondrial antibodies; ANA, anti-nuclear antibodies; ASGPR, asialo-glycoprotein-receptor; BMI, body mass index; CT, computed tomography; CYP, Cytochrome P450; HAV, hepatitis A virus; HBc, hepatitis B core; HBV, hepatitis B virus; HCV, hepatitis C virus; HEV, hepatitis E virus; HIV; human immunodeficiency virus; LKM, liver kidney microsomes; LP, liver-pancreas antigen; LSP, liver-specific protein; MRC, magnetic resonance cholangiography; MRT, magnetic resonance tomography; and p-ANCA, perinuclear antineutrophil.

**Table 2:** Copper sources from the natural environment and industry, with proposed impact on human health.

Copper Source	Human Health Risk Issues	Comments	References
Terrestrial and aquatic environment due to anthropogenetic activity are considered as important.	Copper and other heavy metals as environmental pollutants with their toxicological effects in humans mentioned.	Copper with detrimental effects on human health is discussed but lacking quantitative health risk evaluation.	Briffa, 2020 [6]
Municipal solid waste landfill comprising household, healthcare, and industrial waste.	Copper and other heavy metal accumulation found in water, soil, and plants of municipal solid waste landfill in Vientiane, Laos.	Leaching from landfills, many compounds can harmfully influence human health but lacking quantitative risk data.	Vongdala, 2019 [23]
Plants used as herbal medicines and grown on soils contaminated with heavy metals.	Heavy metals found as potential hepatotoxins in herbal medicinal products may impair human health.	It remains to be clarified how often contaminated herbal medicinal products can trigger HILI.	Quan, 2020 [24]
Heavy metals with wide distribution in the environment due to multiple industrial, technological, medical, agricultural, and domestic applications. Because even low levels of exposure may already be hazardous to humans, global public health concern exists.	Concern of heavy metal toxicity and their potential effects on human and public health is discussed. As systemic toxicants, they are known to cause multiple organ damage, considering variability of molecular mechanisms of toxicity, genotoxicity, and specifically carcinogenicity.	Metallic elements are classified as known or probable human carcinogens according to the US Environmental Protection Agency, and the International Agency for Research on Cancer. Carcinogenicity involves many mechanistic steps, some of which are not clearly elucidated or understood.	Tchounwou, 2012 [29]
Copper–chromium–arsenic (CCA), which is a preservative substance used in various countries to treat timber. This leads to the presence of copper pollution in the soil.	Copper and other heavy metals are widely found and distributed in the environment, raising concerns over their potential effects on human health with impact that may depend on possible routes of heavy metal exposure.	Several studies have shown that toxic metal exposure causes long-term health problems in humans. Little is known about the health impact of mixtures of these toxic elements with additive or synergistic effects.	Witkowska, 2021 [44]
Copper and various other heavy metals are found in herbal medicinal plants due to environmental heavy metal pollution of the soil where the plants grow.	New analytical methods may help detect heavy metals in medicinal plants. Using medicinal plants polluted by copper significantly threatens the health of consumers.	Health hazards are not specified in detail, clearly a difficult approach due to plant contamination by many other heavy metals apart from copper.	Guo, 2023 [48]
Critical evaluation of human health issues by dietary copper. Gaps prevail as well as unresolved issues on health risks due to copper intake.	Results from observation and intervention studies do not support a link between copper and a risk of cardiovascular disease, cognitive decline, arthritis, or cancer.	High amounts of ingested copper seemingly show no causal relationship for various diseases, a discussion that certainly needs further studies for confirmation.	Bost, 2016 [57]
Sources of copper in the environment may include copper water pipes, drinking water, copper cookware, birth control pills, copper intrauterine devices, and fungicides.	Occupational exposure to machinists, plumbers, welders, and others who work with copper are at risk for copper toxicity. Due to tissue injury, disrupted homeostasis is associated with a number of diseases.	By overwhelming body antioxidant systems and inducing DNA damage, ROS finally may lead to degenerative diseases, cancer, cardiovascular disease, and chronic inflammation.	Gaetke, 2014 [61]
Copper found in soil and medicinal herbs surrounding ponds on agricultural land.	Contaminated with heavy metals, herbs growing on such soils can become harmful to living organisms.	Specific risk issues of human health due to contaminated plants were not studied.	Malinowska, 2017 [62]
Urban vehicle traffic contamination by copper and various other heavy metals was studied in road sweeping and waste bottom sediments of retention tanks.	Copper is among the most documented elements on the surface of the street. Human health risk and non-carcinogenetic and carcinogenetic risk was calculated.	Whether data obtained following calculation represent real world conditions remains to be established. Health risk of environmental copper was discussed.	Nawrot, 2020 [65]
High fungicide-derived copper levels detected in soil and vegetation in Owena cocoa (Theobroma cacao L.) plantations in Nigeria raise health concern.	High contaminating copper content in Owena cocoa explains high copper values in commercial chocolate. Local conditions may not be safe for human health.	Due to increased copper levels, use of chocolate is not recommended in patients with copper-related Wilson disease. Local concern for health was not further detailed.	Adeyeye, 2006 [68]
Copper is widely used in the industry and agriculture.	Elevated copper levels are risky as commonly found in the biosphere.	Specific human health concerns are not detailed.	Flemming, 1989 [69]
Overview of the copper situation and broad usage in viticulture areas.	Copper-containing products are used as fungicides and increase environmental intake of copper.	Exposed organisms are at higher risk of suffering from developmental and reproductive disorders.	Kovačič, 2013 [70]
Attic dust studies show historical air pollution by copper and other heavy metals.	Question was raised on potential health hazard posed by copper and other heavy metals as urban pollutants.	Specific risks of human health were not studied in this otherwise pioneering work.	Gaberšek, 2022 [71]
Copper environmental toxicology is of major concern for humans, animals, and plants.	Worldwide contamination of agricultural lands by copper and other heavy metals is viewed as risky.	It has become a serious threat to humans and animals via their entry in the food web.	Rehman, 2019 [74]
Currently, Brazil is among the largest copper ore producers.	Impact of copper mining waste in the Amazon on risks to human health.	Human health risks were not provided in sufficient detail.	Covre, 2022 [75]
Copper uptake by edible plants from soil contaminated with this heavy metal determines potential health risk associated with the ingestion of contaminated food.	Health risk assessment is essential due to copper build-up in plant food and consumption of foods rich in copper. Concern has been expressed that copper can induce serious health disorders.	Environmental copper as part of the soil–plant transfer within the food chain that potentially reaches humans and may lead to major health risks without delineating details of possible diseases.	Shabbir, 2020 [76]
Overview of various environmental patterns and dynamics due to copper that helps examine current human exposures.	High levels of copper can be detrimental to life. More specifically, human health issues related to copper intoxications as exemplified by Wilson disease.	Environmental copper polluting the food chain will impair human health in susceptible individuals due to genetic aberration like in Wilson disease.	Georgopou-los, 2001 [77]
Environmental copper partially may be linked to Alzheimer’s disease development through the food chain.	Dyshomeostasis of copper and its valency in the body, instead of its accumulation, are major determinants for Alzheimer’s disease.	Copper is incriminated as potential major causative factor in the development of Alzheimer’s disease.	Hsu, 2018 [78]
Copper bioavailability, uptake, toxicity aspects and tolerance in plants are reviewed in detail and seen as connected to human health.	Copper in excess has detrimental impact on human health and the prescribed dose–response curve of copper in humans is U-shaped.	Excessive copper levels are viewed as risks to human health and may lead to liver and Alzheimer’s disorders.	Kumar, 2021 [79]
Micronized copper as wood preservatives are potential health risks but need further studies for confirmation.	During decomposition of treated wood, copper-based nanoparticles may be inhaled and can cause harm to human health.	Nanoparticles based on copper could become a potential risk for human health secondary to their inhalation.	Civardi, 2015 [80]
Leachability features of copper and related heavy metals cause health risk in copper mining-impacted sediments.	The hazard index and carcinogenetic risk indices showed significant risks of human exposure, but additional information was not provided.	The carcinogenetic risk of copper must be evaluated under real-world conditions among individuals confronted with copper exposure.	Yan, 2020 [81]
Essentiality and toxicity due to copper was assessed with focus on human health risk including diseases.	A conceptual framework for this type of risk evaluation was applied for analysis of the potential human toxicity by high copper intake.	Human health risks attributable to excess copper intake were discussed and require confirmation.	Stern, 2010 [82]
Potential human health risks of heavy metals in soils exist in copper mining areas.	Copper may promote the formation of amyloid plaques characteristic for Alzheimer’s disease.	Copper together with iron causes atherosclerosis and neurodegenerative diseases.	Filimon, 2021 [83]
The potential exposure and hazards of copper nanoparticles were critically reviewed. Copper nanoparticles are increasingly used, including in cancer chemotherapy.	Copper nanoparticles deposit in both the upper and lower respiratory tract. Prolonged persistence leads to enhanced oxidative stress and inflammatory response due to local irritation.	Current evidence that copper nanoparticles may harm human health is not available, but their toxicology risk is assumed to be ten times lower compared with other copper forms.	Ameh, 2019 [84]
A systematic review and meta-analysis evaluated toxic metal contaminants of the environment and their risk of cardiovascular disease and coronary heart disease.	Exposure to environmental copper in high amounts is significantly associated with an increased relative risk of both cardiovascular disease and coronary heart disease through increased systolic blood pressure.	The increased risk also was attributed to oxidative stress by generation of ROS and a copper–homocysteine complex responsible for vascular injury and endothelial dysfunction.	Chowdhury, 2018 [85]
Health risk assessment of copper was done in soils contaminated with municipal wastes.	Copper with elevated levels can cause respiratory problems, dizziness, nausea, and diarrhea.	Copper is considered in the present study as not a potentially toxic element.	Gujre, 2021 [86]
Implications for setting regulatory health criteria for ingested copper.	Human health risks due to exposure to copper are described and proposals for its safe use are given.	There is good news, as copper is not viewed as a carcinogenetic element, important for clinicians.	Taylor, 2020 [87]
Human health risk of copper was assessed in soil and plants near to a copper smelter, providing high copper values.	Soil samples display a significant level of copper enrichment but the hazard index for non-carcinogenic copper is low in children and adults.	Health risks of copper contaminating the soil near copper smelters are seemingly low despite high copper values in local soil.	Nematollahi, 2020 [88]
Distribution, sources and health risks of heavy metals were assessed in indoor dust across China.	Copper was identified as a contaminant in indoor dust, an important finding as most people spend up to 90% of their times indoors.	No considerable non-carcinogenic risk was found for copper that contaminated indoor dust in Chinese households.	Wang, 2023 [89]
Environmental health hazards of e-cigarettes and their components: oxidants and copper in e-cigarette aerosols.	Copper is among other elemental constituents identified in aerosol volumes from disposable e-cigarettes.	E-cigarette aerosols have a lower health risk than conventional cigarette smoke as copper amount is smaller.	Lerner, 2015 [90]
Association between serum copper levels and lung cancer risk was studied in a meta-analysis	Serum copper levels are higher in patients with lung cancer in comparison with a control group lacking lung cancer.	Such correlation between serum copper levels and cancer does not allow for assuming a priori any firm causality association.	Zhang, 2018 [91]

Abbreviations: HILI, herb-induced liver injury; ROS, reactive oxidative species.

**Table 3:** Modified Leipzig scoring system for Wilson disease.

Parameter	Score
**Kayser–Fleischer rings** Present Absent	2 0
**Serum ceruloplasmin** >20 mg/dL (normal) 0–5 mg/dL 6–11 mg/dL 11–20 mg/dL	0 3 2 1
**24 h urinary copper (in the absence of chronic cholestatic liver disease)** >100 µg 40–100 µg <40 µg	2 1 0
**Coombs-negative hemolytic anemia with liver disease** Present Absent	1 0
**Mutational analysis** On both chromosomes detected On one chromosome detected No mutation detected/test not done	4 1 0
**Liver biopsy for histology suggestive of Wilson disease with** Orcein- or rhodamine-positive granules	1
**Neurobehavioral symptoms** Present Absent	2 0
**Typical features on magnetic resonance imaging of the brain** Present Absent	1 0
**History of Wilson disease in a family member** Sibling death from liver disease/neurological disease suggestive of Wilson disease	1
**Evaluation** Diagnosis established Diagnosis possible, more tests needed Diagnosis very unlikely	Total score ≥4 3 ≤2

**Table 4:** Iron sources from the natural environment and industry, with proposed impact on human health.

Iron Source	Human Health Risk Issues	Comments	References
Iron overload is possibly related to human neurodegenerative disorders.	Evidence-based data in support of this assumption were not provided and need to be supplemented.	It is certainly challenging for future studies to close up the gap between this disease and excess iron.	Abbaspour, 2014 [183]
Iron homeostasis is viewed as disrupted upon exposure to various environmental pollutants and may have health effects.	Environmental pollutants may give rise to disordered iron homeostasis, which could lead to cytotoxicity and increased cancer risk in exposed humans.	Firm evidence that this proposal is functioning under normal field conditions and is lacking requiring additional studies for verification.	Guo, 2015 [193]
Iron in humans with disturbed cellular iron homeostasis caused by pollutants in the environment led to increased intestinal iron uptake from ingested food.	This ultimately was linked to human diseases like diabetes mellitus, cancer, and renal, cardiovascular, respiratory, autoimmune, neurodegenerative (like Alzheimer’s disease), and cerebrovascular diseases.	Challenging proposal that disturbed iron homeostasis may increase the risk cancer incidence and cancer mortality, but further studies are required to prove this concept.	Schreinema-chers, 2016, [194]
Human health hazard assessment of iron intake with arsenic-safe ground water in Jashore, Bangladesh.	The assessment revealed that the non-carcinogenetic risks due to ingestion of iron were 1.446 for adults and 0.590 for children.	Regretfully, iron content in exposed individuals was not measured, though viewed as a risk for heart disease and diabetes.	Gosh, 2020 [195]
Iron status and iron homeostasis are often environmentally altered and are risk factors for human health.	Tissue-specific brain iron overload is observed in degenerative neurological diseases without an increase in systemic iron.	A gap in understanding exists for brain overload despite a lack of increase in systemic iron stores, more evidence is needed.	Lal, 2020, [196]
Industry-relevant iron and iron status have been associated with attention deficits.	Higher iron concentrations and ferritin were jointly associated with worse attention behaviors.	Using data on ferritin as a diagnostic parameter of iron body stores remains problematic as it is not specific.	Schildroth, 2024 [197]
Presence of abundant iron-rich air pollution nanoparticles, emitted from industry and sources related to traffic likely represents a major health risk for cardiac disease with co-associated metals.	Exogenous nanoparticles (with diameter around 15–40 nm) within myocardial mitochondria of young, highly exposed individuals are dominantly iron-rich, eventually causing cardiovascular disease.	A cautionary note with focus on confounding variables is warranted and may include other trace exogenous metal species like aluminum and titanium as co-associated with these iron-rich nanoparticles.	Maher, 2020 [198]
Iron loading may be associated with various health hazards as exemplified by a high number of assumed diseases. Iron may be inhaled by industrial workers: iron miners, foundry workers, or welders.	Limited data exist on the role of ferriferous materials inhaled by industrial workers as a potential risk factor in causing pulmonary tract neoplasms and the dissemination of inhaled iron via macrophages to other body tissues to result in systemic iron loading.	Assuming the exposed workers were healthy before and have an iron homeostatic system that is not impaired, it should be no problem for these workers to limit iron uptake and remove excess iron.	Weinberg, 2010 [199]
Iron ore mining in Pahang, Malaysia, with potential human health risks of this special heavy metal detected in some surface soils.	For iron, no significant potential health risk was found to children and adults as the hazards indices of non-carcinogenic risks all were lower than one.	Data are as expected. If heathy individuals did incorporate excessive iron, they should have a system allowing for iron homeostasis.	Diami, 2016 [200]

**Table 5:** Cadmium sources from the natural environment and industry, with proposed impact on human health.

Cadmium Source	Human Health Risk Issues	Comments	References
Derived from industrial as well as agricultural sources, exposure to cadmium primarily occurs though the ingestion of food and water contaminated with cadmium.	Epidemiological data relate both environmental and occupational cadmium exposure to various types of cancer, including breast, nasopharynx, lung, kidney, prostate, and pancreas.	The liver and kidneys are extremely sensitive to the toxic effects of cadmium, likely due to the ability of these organs to form metallothioneins that bind tightly the toxic cadmium ions.	Genchi, 2020, [263]
High rates of soil to plant transfer renders food contaminated with cadmium as the main source of exposure among non-smoking and non-occupationally exposed individuals.	In prospective studies in Japan and the US an excess of cancer mortality was found to be associated with environmental cadmium exposure. In addition, cadmium may cause endometrial cancer.	A very large health risk is seemingly associated with cadmium exposure at levels experienced by many populations in the world. No escape is possible as cadmium is found to be ubiquitous.	Satarug, 2011 [268]
Cadmium derived from the environment exerts toxic effects on various organs and in addition, is classified as a human carcinogen.	Toxicity primarily relates to kidneys, bones, and the respiratory system, while cancer may develop in lungs and prostate due to alteration of DNA repair.	A variety of risk mitigation recommendations have been presented but their efficiency remains unclear due to multiple cadmium sources and its ubiquity.	WHO, 2024 [274]
Cadmium is naturally found in soil, minerals, and water and enters the human body via the lungs and the digestive tract. Smokers are especially at risk as tobacco leaves are often contaminated by cadmium derived from contaminated soil.	Cadmium is described by both its toxicity and health effects. Emphasis is given to high blood pressure and lung diseases like asthma and emphysema. It may play a crucial role in the development of Alzheimer’s disease, multiple sclerosis, Parkinson’s disease, and Huntington’s disease.	A cautionary statement is required with respect to the lung diseases also triggered by smoking itself, which must be viewed as a confounding variable. Regarding the mentioned neurology diseases, there are likely further studies required for confirmation of a causal attribution.	Charkiewicz, 2023 [275]
Various environmental sources of cadmium are mentioned. The blood concentration of cadmium serves as a reliable indicator for a recent exposition as opposed to the urinary concentration, which reflects past exposure.	Several studies showed cadmium association with bone injury. Cadmium was also implicated in the Itai-Itai disease, characterized by osteoporosis and a high rate of fractures, caused by use of rice contaminated with cadmium following cadmium irrigation.	Also under discussion is the role of cadmium in causing cancer, including renal cancer, if cadmium specifically enters the human body via the respiratory system, but molecular mechanisms of carcinogenesis by cadmium are unknown.	Godt, 2006 [276]
Cadmium exposure is widespread among non-occupationally exposed populations and non-smokers.	A recent systematic review revealed that cadmium exposure potentially leads to deteriorations of cognitive abilities.	This topic is still under discussion as only 10/15 eligible studies supported such association while 5/10 studies did not.	Chatterjee, 2022 [277]
Cadmium exists in the environment due to use of fossil fuels, metal ore combustion, and waste burning.	Cadmium is a health risk. Cadmium only injures cells which cannot synthesize metallothioneins capable of scavenging free radicals.	Instead, metallothioneins formed in cells prevent cellular cadmium toxicity because toxic radicals are effectively scavenged.	Rafati Rahimzadeh, 2017 [278]
Cadmium is regularly found with other heavy metals such as zinc, copper, and lead in the polluted environment.	Neurologic dysfunction can occur as parkinsonism, impaired higher cortical functioning, and olfactory disturbances.	Divergent statements: Liver injury after exposure to cadmium was not reported by some [269,279], but was reported by others [280].	Koons, 2023 [269]; Hong, 2021 [279]; Ikeda, 1997 [280]
Cadmium present in the environment and causing health hazards in the US population was examined in a survey from 1999 to 2018.	Cadmium is toxic to human health, and upon exposure, it significantly increases overall mortality including cancer deaths and deaths from causes related to cardiovascular disease.	The mediating effect of smoking was estimated at 32%, as opposed to a large proportion of 68%, for which a direct effect of cadmium remained.	Moon, 2023 [281]

**Table 6:** Arsenic sources from natural environments and industry, with proposed impact on human health issues.

Arsenic Source	Human Health Risk Issues	Comments	References
Exposure to toxic arsenic in tap water worldwide.	Cancers of the skin and various internal organs as well as noncarcinogenic effects were reported.	This early report described major health hazards.	Abernathy, 1999 [333]
Humans are at risk of exposure to arsenic through air, food, or water.	Health hazards include vascular changes, diabetes, and cancers of organs such as the bladder, lung, liver, kidney, and prostate.	This, again, is one of the earlier reports on health issues.	Abernathy, 1999 [334]
Arsenic from natural sources causes global health problems.	Arsenic causes cancers of the skin, lung, and bladder and many disorders of various organs and tissues unrelated to cancer. They all may impair health.	Summary of health hazards due to prolonged arsenic uptake.	Ng, 2003 [335]
Arsenic exposure may occur near factories that produce metals.	General health hazards include higher mortality rates due to cancers of the skin, lung, liver, urinary bladder, kidney, and colon.	Arsenic causes a bundle of health hazards including cancer.	Tchounwou, 2003 [336]
Arsenic contaminating drinking water was assessed for health effects on some liver tests and histology.	Patients (93/193) with hepatomegaly showed elevated ALT (>40 U/L), AST (>40 U/L), and ALP (>400 U/L). Liver histology showed portal fibrosis in 91.3% of cases, cirrhosis in 2.9% of cases, and a normal picture in 5.8% of cases.	Such high values of increased liver test incidence have not been reported in other previous reports on this topic.	Guha Mazumder, 2005 [310]
Groundwater polluted by arsenic in India.	Skin lesions like cancer, gangrene, and melanosis. Spontaneous abortion.	All depended on arsenic levels or exposure lengths.	Mukherjee, 2005 [337]
Arsenic sources are water, air, and food.	Health hazards include lung cancer and fetal loss.	Data were obtained prior to regulatory restrictions.	Kapaj, 2006 [338]
Arsenic is a major environmental pollutant as a contaminant of drinking water.	Chronic arsenic exposure in humans results in malignant, degenerative, and inflammatory changes.	Health hazards affecting many organs and tissues were reported and analyzed in detail.	Singh, 2007 [339]
Inorganic arsenic is a human carcinogen in adults and also a general toxicant even for fetuses.	Inorganic arsenic and its several metabolites pass through the placenta and may increase the risk of fetal loss and retard fetal growth.	Arsenic is a major health hazard for adults as well as for fetuses, and a challenging health issue.	Vahter, 2008 [340]
Arsenic found in wells along river Indus in Pakistan leads to health risks.	Prevalence of arsenicosis skin lesions overall was 13.5%, as assessed in 72/534 cases.	Prevalence rates were highest at 100–199 ppb arsenic levels.	Fatmi, 2013 [341]
More than 200 million persons worldwide might be chronically exposed to arsenic tap water.	Substantial health issues of prolonged exposures to arsenic generally relate to skin and neurological diseases, in addition to malignant tumors.	For some cancer types, the risk is increased in a dose-dependent linear trend.	Naujokas, 2013 [342]
Two consecutive endemic long-term exposures to arsenic from drinking water in Taiwan.	Several diseases have been well documented to be associated with chronic consumption of drinking water that contained arsenic as pollutant contaminant.	Illnesses related to the consumed arsenic partially showed a dose–response relation.	Chen, 2014 [343]
Arsenic polluted water through natural and anthropogenic sources like mining, industrial processes, and the production and use of pesticides.	Arsenic exposure is known to be associated with the development of vascular diseases including stroke, ischemic heart disease, and peripheral vascular disease. Similarly, and according to the International Agency for Research on Cancer and the US FDA, there is an increased risk of tumors of the bladder, lungs, kidneys, and liver.	A critical comprehensive overview was provided on health hazards due to arsenic exposure.	Palma-Lara, 2020 [344]
Contamination of food by arsenic is seen as a public health issue in the Middle East, where the food supply relies on its import.	Exposure to arsenic leads to an increased risk of diseases such as dysbiosis, obesity, metabolic syndrome, diabetes, chronic kidney disease, chronic heart disease, cancer, and maternal as well as fetal complications.	An informative overview on many diseases caused by prolonged exposure to arsenic was provided.	Khan, 2022 [345]
Humans generally become exposed to arsenic by contaminated tap water.	Exposure to arsenic can result in many health problems, ranging from cancer to skin diseases. It may cause genotoxicity and lead to life-threatening abnormalities of the inflammatory and immune system.	The focus was on new insights on genotoxicity, and epigenomic changes and other alterations.	Ozturk, 3022 [346]
Exposure to arsenic may occur via use of contaminated drinking water and food.	In patients from India with gallbladder carcinoma, a high arsenic concentration was found in blood and bile samples, as well as in biological gallbladder tissue, gallbladder stones, and hair samples.	New insights on a high gallbladder cancer risk due to arsenic intake were reported.	Kumar, 2023 [347]
Around 80 million people in India consume arsenic contained in groundwater.	In India, more than 0.1 million deaths and 0.3 million cases of illness are due to arsenic-contaminated groundwater. Arsenic in utero is associated with impaired cognitive development.	Arsenic damages chromosomal deoxyribonucleic acid (DNA).	Aryan 2024 [348]
Arsenic contaminating the drinking water in Ethiopia was studied.	The mean of arsenic concentration in the groundwater samples was 11.15 ± 9.38 µg/L. The cancer risk for children was 1.15 × 10^−2^ and the risk for adults 4.95 × 10^−3^.	The cancer risks exceed commonly accepted threshold values.	Demissie, 2024 [349]
Long-term use of arsenic in water is potentially life-threatening.	The estimated health risk of arsenic in drinking water was up to 1.63 × 10^−6^ disability-adjusted life years (DALYs) in some parts of Northern China.	Prolonged arsenic uptake in drinking water can cause increased DALYs.	Dou, 2024 [350]
EFSA updated its risk assessment on arsenic in food.	Epidemiological studies show that chronic intake is associated with increased risk of cancers of the skin, bladder, and lung.	Epidemiology, again, ascertained increased cancer risks.	EFSA, 2024 [351]
Arsenic has the ability to move in environmental media and has become a major public and environmental concern.	Hypertension and atherosclerosis are the most extensively studied topics, with redox imbalance, apoptosis, and methylation being the primary mechanistic clues. Cardiovascular damage caused by arsenic includes arrhythmia, cardiac remodeling, vascular leakage, and abnormal angiogenesis.	This article offers a comprehensive overview of current research and actual data on cardiovascular hazards caused by arsenic.	Han, 2024 [352]
The association between higher but not lower arsenic levels in drinking water and lung cancer is well described.	Individuals exposed to low to moderate levels of arsenic (<150 μg/L) were at an elevated risk of developing or dying from lung cancer. For lung cancer incidence, the predicted posterior mean relative risks (RRs) at arsenic concentrations of 150 μg/L were 1.11 (0.86–1.43).	Information was presented on the effect of exposure to low and moderate arsenic levels on outcomes of lung cancer.	Issanov, 2024 [353]
Vegetables can accumulate high arsenic amounts and are widely consumed.	Health risk assessments associated with arsenic exposure through consumption of water spinach and amaranth were conducted using prediction models and soil samples collected in Taiwan.	Total contents of arsenic in soil were predictors of arsenic amounts in water spinach.	Liao, 2024 [354]
Though findings were still vague, some evidence supported that arsenic exposure contributes to non-alcoholic fatty liver disease (NAFLD) risk.	NAFLD was diagnosed by liver ultrasound, and logistic regression was used to evaluate the associations. The results suggested that inorganic arsenic exposure is positively associated with NAFLD risk, whereby efficiency of arsenic methylation plays a role in the NAFLD.	Clues were presented to explore potential interventions for the prevention of NAFLD.	Liu, 2024 [355]
Arsenic pollution causing severe health issues is widely reported and has gained global attention in the last few decades.	Bibliometric analysis of arsenic pollution and its health hazards has revealed that arsenic pollution is primarily caused by anthropogenic sources, and the key sources of arsenic exposure are drinking water, sea food, and agricultural products. Arsenic pollution is related to major health hazards such as cancer.	Arsenic has a biogeochemical cycle that, with its complexity, plays a significant role in pollution and thus in emerging health problems.	Sevak, 2024 [356]

Abbreviations: ALP, alkaline phosphatase; ALT, alanine aminotransferase; AST, aspartates aminotransferase; EFSA, European Food Safety Authority; FDA, Food and Drug Administration.
